# MAGI3 deficiency unleashes β-catenin conformational change to drive metastatic progression and mTOR inhibitor resistance in ccRCC

**DOI:** 10.1038/s41419-026-08563-x

**Published:** 2026-03-24

**Authors:** Siyu Gu, Haibo Wang, Hua Liu, Yumeng Yang, Yu Guo, Pengyan Fa, Lijie Zhang, Yang Yang, Xuan Qi, Qiong Qin, Ran Song, Xiaomei Yang, Junqi He

**Affiliations:** 1 Department of Biochemistry and Molecular Biology, School of Basic Medical Sciences, Capital Medical University, Beijing, China; 2 Laboratory for Clinical Medicine, Capital Medical University, Beijing, China; 3 Beijing Laboratory of Oral Health, Capital Medical University, Beijing, China; 4 Center for Endocrine Metabolism and Immune Diseases, Beijing Luhe Hospital, Capital Medical University, Beijing, China

**Keywords:** Tumour-suppressor proteins, Cancer therapeutic resistance, Renal cell carcinoma, Ubiquitylated proteins

## Abstract

Metastatic clear cell renal cell carcinoma (ccRCC) remains lethal due to therapy resistance, and while dysregulated Wnt/β-catenin signaling drives progression, its post-translational regulation is poorly understood. Through multi-omics analysis of TCGA/GEO datasets, we identified MAGI3 as a key metastasis suppressor in ccRCC. Functional validation revealed that MAGI3 loss enhances invasion, migration and metastatic potential in vitro and in vivo. Mechanistically, MAGI3 binds β-catenin’s C-terminus via PDZ domains, disrupting intramolecular N-terminus–ARM domain interactions to expose phosphorylation sites, thereby enabling GSK-3β–mediated β-catenin phosphorylation and ubiquitin-dependent degradation. Critically, low MAGI3 hyperactivates β-catenin and drives mTOR inhibitor resistance. Combining Everolimus with the Wnt inhibitor XAV-939 slashed viability and invasion in resistant cells. Clinically, patients whose tumors exhibited high MAGI3 and low β-catenin expression demonstrated significantly improved response to Everolimus therapy. In conclusion, MAGI3 is a critical gatekeeper of β-catenin destruction in ccRCC. Its loss defines a metastatic, therapy-resistant subtype targetable by dual mTOR/Wnt blockade. Therefore, MAGI3 expression may stratify patients for personalized therapy.

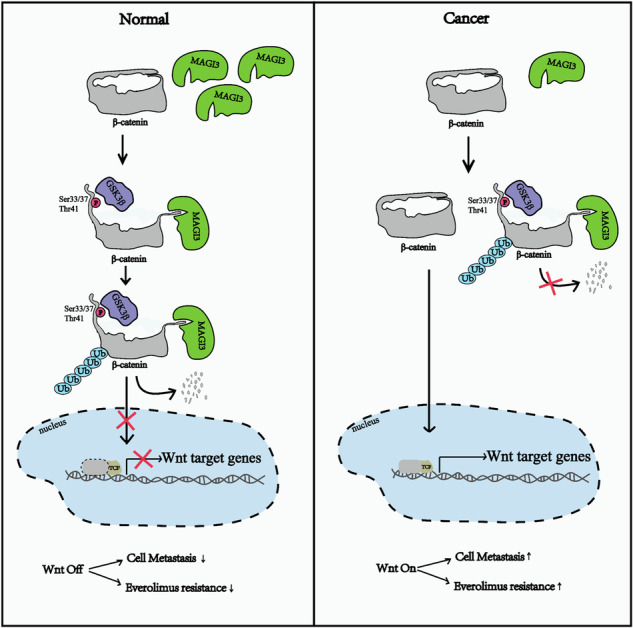

## Introduction

Renal cell carcinoma (RCC) represents as a lethal urological malignancy, where the clear cell subtype (ccRCC) dominates ~70% of cases and drives disproportionate mortality [1]. While diagnostic and therapeutic strategies have advanced, the incidence of metastatic RCC (mRCC) paradoxically appears to be rising—potentially reflecting improved detection or other factors [2].

While surgical resection remains curative for localized disease, 20–40% of patients have distant metastases at diagnosis [3], additionally, approximately 25% of initially localized cases progress to mRCC following local therapy, which historically demonstrates a median survival of just 12 months [4]. The therapeutic landscape for advanced ccRCC remains challenging, as most patients show a limited response to conventional chemotherapy radiotherapy, and first-generation cytokine therapies (IL-2 or IFN-α) [5].

Dysregulation of the von Hippel-Lindau (VHL)-hypoxia-inducible factor (HIF)-vascular endothelial growth factor A (VEGFA) axis is a cornerstone of ccRCC pathogenesis [6, 7]. Somatic VHL inactivation occurs in over 90% of sporadic ccRCC cases [8, 9], leading to constitutive stabilization of HIF-2α drives the persistent upregulation of angiogenic factors and activates key oncogenic pathways, including the RAS/RAF/MAPK and PI3K/AKT/mTOR cascades [10]. Therapeutically targeting these vulnerabilities has led to the development of agents such as tyrosine kinase inhibitors (TKIs), mTOR inhibitors, and more recently, the HIF-2α inhibitor Belzutifan [11, 12]. These advancements have progressively improved patient outcomes, with current frontline combination therapies extending median overall survival beyond 30 months in clinical trials [13, 14]. Despite this progress, significant clinical challenges persist. Primary resistance affects 20–30% of patients, and the majority of initial responders invariably develop acquired resistance within 6–15 months [15]. Consequently, metastatic ccRCC remains largely incurable, with a dismal 5-year survival rate of approximately 12% [16]. Therefore, a deeper understanding of the molecular mechanisms underlying therapy resistance is imperative for developing more effective treatment strategies and improving long-term survival for ccRCC patients.

Lines of evidence report that dysregulated Wnt/β-catenin signaling associates with cancer development and progression, particularly in ccRCC [17]. While aberrant Wnt/β-catenin activation is well-documented in RCCs broadly [18, 19], its precise role in ccRCC reveals conflicting reports and mechanistic uncertainties. Genetic silencing of β-catenin or pharmacological inhibition of the Wnt/β-catenin pathway effectively suppresses cancer cell proliferation, induces apoptosis, and overcomes chemoresistance in various cancers [20]. Yet, ccRCC typically displays low β-catenin mRNA levels [21] and infrequent mutations in the β-catenin gene itself [22]. Intriguingly, cytoplasmic accumulation of β-catenin protein has been identified as an independent risk factor for cancer-specific survival in ccRCC patients [23]. This finding suggests unique post-translational regulatory mechanisms, further complicated by the absence of APC mutations typically associated with β-catenin stabilization in other cancers [24].

Our analysis of TCGA and GEO data showed that MAGI3 expression is significantly reduced in mRCC, and this reduction correlates with poorer clinical outcomes. Functional studies establish MAGI3 as a novel tumor suppressor in RCC, suppressing tumor cell migration and invasion. Mechanistically, MAGI3 destabilizes β-catenin by altering its intramolecular conformation. This structural change allows GSK-3β to phosphorylate β-catenin, marking it for destruction by the proteasome. Crucially, MAGI3-mediated suppression of Wnt signaling sensitizes ccRCC to mTOR inhibitors. Therefore, dual inhibition of mTOR and Wnt signaling synergistically suppresses tumor growth.

Our findings establish dual mTOR and Wnt pathway modulation as a promising new therapeutic approach for advanced ccRCC, and highlight MAGI3 as a potential predictive biomarker to guide personalized treatment strategies.

## Materials and methods

### Ethics statement and patient samples

This study utilized two independent cohorts of ccRCC patients, approved by the Ethics Committee of Capital Medical University (Approval No. 2015SY08). All procedures were conducted in accordance with the Declaration of Helsinki, and written informed consent was obtained from all participants prior to sample collection.

Cohort 1: Primary Tumor Cohort. This cohort consisted of patients who underwent curative-intent radical or partial nephrectomy for ccRCC. The cohort included both patients with localized disease and those with synchronous metastases at diagnosis. Formalin-fixed, paraffin-embedded (FFPE) tumor tissues from the resected primary renal tumors were collected. The primary objective of analyzing this cohort was to evaluate the protein expression level of MAGI3 and β-catenin in primary ccRCC and its correlation with clinicopathological features and metastatic status.

Cohort 2: Everolimus-Treated metastatic cohort comprised patients with metastatic ccRCC (mRCC) who had progressed following prior systemic therapy and subsequently received Everolimus treatment. Patient inclusion criteria were as follows: (1) Histologically confirmed ccRCC with documented disease progression after prior lines of systemic therapy (consistent with standard sequencing where Everolimus is used post-tyrosine kinase inhibitors and/or immunotherapy). (2) Initiation of oral Everolimus therapy (10 mg/day) as a monotherapy for mRCC. (3) Availability of pre-Everolimus treatment FFPE tumor specimens (obtained from prior nephrectomy). (4) Availability of complete radiological and clinical follow-up data to assess therapeutic response to Everolimus. Exclusion Criteria: (1) Concurrent receipt of any other antitumor therapies during the Everolimus treatment period. (2) Discontinuation of Everolimus before completing one full treatment cycle (approximately 4 weeks) due to intolerable adverse events or patient non-compliance. (3) Inadequate tumor tissue for immunohistochemical analysis. Response to Everolimus was evaluated by contrast-enhanced computed tomography (CT) scans performed at baseline and at regular intervals during treatment. Responses were categorized according to Response Evaluation Criteria in Solid Tumors version 1.1 guidelines into: Complete Response (CR), Partial Response (PR), Stable Disease (SD), and Progressive Disease (PD). For subsequent biomarker correlation analysis, patients were dichotomized into a disease control group (SD + PR) and a progressive disease group (PD).

### Data sets collection

RNA sequencing data and clinical annotations for ccRCC were obtained from The Cancer Genome Atlas (TCGA) via Synapse (http://synapse.org). Additional microarray datasets—GSE12606, GSE31232, GSE36895, GSE73731 and GSE22541—were downloaded from the GEO database (https://www.ncbi.nlm.nih.gov/geo/).

### Cell culture, transfection, and plasmids

Human renal cancer cell lines 769-P (CLS Cat# 300106/p490_769-P, RRID: CVCL_1050), 786-O (RRID: CVCL_1051), and 293T (DSMZ Cat# ACC-305, RRID: CVCL_0045) were obtained from ATCC. Cells were maintained at 37 °C in 5% CO₂ in DMEM (for 293T) or RPMI-1640 (for 769-P and 786-O), supplemented with 10% FBS and 1% penicillin-streptomycin.

293T cells were transfected using polyethyleneimine (PEI; Polyscience) (#23966, Polyscience, IL, USA), while 786-O and 769-P cells were transfected with Lipofectamine 3000 (#L3000-015, Invitrogen, CA, USA) for siRNA or plasmid delivery. For stable transfection, cells were selected with 1000 µg/mL G418 (#S3028, Selleck, TX, USA) or 5 µg/mL puromycin (#p8833, Sigma, MO, USA) after introducing MAGI3 expression plasmids or lentiviral shRNA particles (#sc-42004-V, Santa Cruz, CA, USA).

All cell lines were authenticated by short tandem repeat (STR) profiling within the last 3 years and tested negative for mycoplasma contamination.

Constructs including pEGFP-N4-MAGI3 and pcDNA3-V5/His-MAGI3 were provided by Dr. Randy Hall (Emory University). Flag-tagged β-catenin constructs and mutants were gifts from Dr. Wei Wu (Tsinghua University). Introduction of the T779A mutation into the Flag-β-catenin construct was achieved through PCR and verified by bidirectional sequencing. The expression vector for GST-β-catenin was constructed via PCR. All constructs were validated by sequencing, with detailed primer sequences listed in Supplementary methods Table 1.

### Transwell migration and invasion assay

For transwell assays, 2 × 10⁴ cells were seeded in the upper chamber (Corning #3422, NY, USA) in serum-free medium. Matrigel (#356234, BD Biosciences, NJ, USA; 1:40 dilution) was used for invasion assays. Medium with 10% FBS was added to the lower chamber. After 17 h, non-migrated cells were removed; filters were fixed in 4% paraformaldehyde and stained with 0.2% crystal violet. The cells on the lower surface of the Transwell chamber were quantified using Image J software, and the results were presented as mean ± standard deviation.

### Wound healing assay

Cells (5 × 10⁵/well) were seeded in 6-well plates. After reaching confluence, a scratch was made using a pipette tip. The cells were washed and cultured in serum-free medium. Images were captured at 0 and 12 h using a phase-contrast microscope.

### IC_50_ determination and drug combination assay

IC₅₀ values were determined by treating cells with increasing concentrations of everolimus (TargetMol, #T1784; 10 nM to 100 μM) for 48 h. Cell viability was assessed using CCK-8 and analyzed with GraphPad Prism.

For combination assays, cells were treated with everolimus (2 μM for 786-O; 1 μM for 769-P) alone or combined with XAV-939 (SelleckChem, #S1180; 5 μM). After 48 h, viability was again assessed using CCK-8. All experiments were performed in triplicate.

### Colony formation assay

Cells were seeded at 500 per well in 6-well plates. The following day, everolimus and XAV-939 were added. Colonies that formed over the ensuing 10–14 days were then fixed with 4% paraformaldehyde, stained with crystal violet, and counted using ImageJ.

### Immunohistochemistry

We carried out immunohistochemical staining using our established protocol [25]. Briefly, tissue sections were incubated overnight at 4 °C with primary anti-MAGI3 antibody (Atlas Antibodies, Cat# HPA007923, RRID: AB_1853503). The following day, sections were incubated for 15 min at room temperature with horseradish peroxidase (HRP)-conjugated secondary antibodies. Signals were visualized using a DAB substrate kit (ZSGB-BIO), and nuclei were counterstained with hematoxylin. After dehydration through agraded alcohol series and clearing in xylene, slides were mounted with resin. Finally, whole slides were scanned at 40× magnification on an Aperio GT450 slide scanner (Leica Biosystems) using ImageScope software (Leica Biosystems) to generate digital images.

The expression levels of MAGI3 and β-catenin were independently evaluated by two board-certified pathologists who were blinded to all clinical and pathological data. Staining intensity was graded on a scale of 0-3 (0, negative; 1, weak; 2, moderate; 3, strong). The proportion of positive tumor cells was scored on a scale of 0-4 (0, 0%; 1, 1–25%; 2, 26–50%; 3, 51–75%; 4, >75%). The final histochemical score (H-score) was calculated by multiplying the intensity grade by the corresponding percentage of positive cells (using the median value of each percentage range: 0, 0%; 1, 12.5%; 2, 37.5%; 3, 62.5%; 4, 87.5%), yielding a theoretical range of 0–12.

### Bioinformatic analysis

We performed Gene Set Enrichment Analysis (GSEA) following established methods [25]. Enrichment scores were calculated using MSigDB database for predefined gene sets, including JAEGER_METASTASIS_UP(M5740) and WILLERT_WNT_SIGNALING (M1805). Statistically significant gene sets met an FDR cutoff of <0.25.

### Co-immunoprecipitation (Co-IP) and western blot analysis

Co-IP and immunoblotting experiments were performed according to established protocols [26, 27]. The following primary antibodies were utilized: MAGI3: sc-136471 (Santa Cruz Biotechnology) and NBP1-81266 (Novus Biologicals), GFP:598 (MBL International), FLAG (M185-3L, MBL International), His (PM032, MBL International), HA (561, MBL International), β-Catenin: Total (#9562, Cell Signaling Technology) and phospho-specific (Ser33/37/Thr41; #9561, Cell Signaling Technology), β-actin: bs-0061R (Bioss), used as the loading control.

### GST pull-down assay

We performed GST pull-down assays using established protocols [28]. GST-fusion proteins were expressed in E. coli BL21 and affinity-purified using glutathione Sepharose beads (Cytiva, formerly GE Healthcare). To capture interacting proteins, cell lysates containing potential binding partners were incubated with the GST-protein-bound beads for 3 h at 4 °C. Following five washes with lysis buffer to remove nonspecific interactions, specifically bound complexes were eluted from the beads for downstream analysis.

### Fluorescence Lifetime Imaging Microscopy-Förster Resonance Energy Transfer (FLIM-FRET)

293 T cells were seeded in 35-mm glass-bottom dishes and transfected the following day using polyethylenimine (PEI) transfection reagent. To interrogate the intramolecular interaction of β-catenin and the impact of MAGI3, the following plasmid combinations were employed: (1) CFP-β-cat-N-tail (negative control), (2) CFP-β-cat-N-tail + YFP-β-cat-ΔNT (interaction test), and (3) CFP-β-cat-N-tail + YFP-β-cat-ΔNT + Flag-MAGI3 (functional test). After 36 h, fluorescence lifetime imaging of live cells was performed using a Nikon AXR NSPARC confocal microscope equipped with a 100× immersion objective. The average fluorescence lifetime (τ) of the CFP donor was calculated from at least 3 individual cells per condition across three independent experiments. A significant decrease in the donor fluorescence lifetime (τ) signifies efficient FRET, indicating close proximity (<10 nm) and thus a direct molecular interaction or conformational change.

### Quantitative real-time-PCR (qPCR)

Total RNA was extracted from cells using RNA-Quick Purification Kit (Yishan Biotechnology, #RN001, Shanghai, China) according to the manufacturer’s instructions. First-strand cDNA was generated with HiScript® III RT SuperMix for qPCR (+gDNA wiper) (Vazyme, #R323, Nanjing, China). qPCR was carried out according to the manufacturer’s instructions (Life Technologies). The gene expression was normalized against β-actin in the same sample. The primers used were listed in Supplementary methods Table 2.

### Immunofluorescence

786-O cells in 6-well plates were by fixation, permeabilization, and co-localization assessment using anti-MAGI3 and anti-β-catenin antibodies. Visualization was achieved through confocal microscopy (Leica DM6000 CS, Germany) after secondary antibody incubation and DAPI staining for nucleus visualization.

### In vivo studies

All animal experiments were approved by the Institutional Animal Care and Use Committee (IACUC) of Capital Medical University (Protocol No. AEEI-2020-133) and conducted in accordance with the relevant guidelines and regulations. Sample sizes for all experiments were determined based on power analysis of preliminary data or prior experimental experience to ensure adequate statistical power. Mice were randomly assigned to experimental groups using a random number generator. Due to the distinct nature of the treatments (e.g., different drug administration protocols), investigators could not be blinded to group allocation during the dosing phase. However, tumor volume measurements and subsequent endpoint analyses were performed by investigators blinded to the group assignments to minimize bias.

Tail Vein Metastasis Model. Female NOD/SCID mice (4–5 weeks old; RRID: BCBC_4612, *n* = 7–8 per group) were injected via the tail vein with 786-O-shCtrl or 786-O-shMAGI3 cells. Mice were euthanized 12 weeks post-injection, lungs were harvested, and surface metastatic nodules were counted. Lung tissues were fixed in 4% paraformaldehyde, embedded in paraffin, sectioned, and stained with hematoxylin and eosin (H&E) for histological confirmation.

Subcutaneous Xenograft and Drug Efficacy Model. 786-O cells with stable MAGI3 knockdown or control were subcutaneously injected into the flanks of 7-week-old male BALB/c nude mice (5 × 10^6^ cells in 100 μL PBS). Once tumors reached a palpable size (approximately 80–100 mm³), mice bearing MAGI3-knockdown (shMAGI3) tumors were randomly assigned to four groups (*n* = 5 per group): vehicle control, Everolimus (5 mg/kg, i.p., every 3 days), XAV-939 (10 mg/kg, i.p., every 3 days), and the combination (XAV-939 administered 30 min prior to Everolimus, same doses and schedule). Treatments were administered for 21 days. Tumor dimensions were measured regularly with calipers, and tumor volume was calculated using the formula: Volume = (Length × Width²)/2. Throughout the treatment period, all mice were closely monitored daily for signs of systemic toxicity and changes in overall health. Key parameters recorded included: Body Weight: Measured and recorded every 3 days concurrently with tumor measurement. A loss of >20% of initial body weight was predefined as a humane endpoint. Clinical Signs: Daily observation for signs of distress, including reduced mobility, hunched posture, ruffled fur, lethargy, and signs of pain. Food and Water Intake: General consumption was qualitatively assessed. Drug Injection Site Reactions: The intraperitoneal injection sites were inspected for signs of inflammation, infection, or ulceration.

Mice were euthanized by CO₂ asphyxiation followed by cervical dislocation when the tumor volume exceeded ~1500 mm³, or if they met any predefined humane endpoint (e.g., severe body weight loss, ulcerated tumors, or significant distress), whichever occurred first. At the endpoint, tumors were excised and weighed.

### Protein structure prediction and molecular docking analysis

We retrieved the predicted three-dimensional structures of MAGI3 and β-catenin from the AlphaFold Protein Structure Database. To investigate the potential interaction between MAGI3 and β-catenin, molecular docking simulations were then conducted to predict the binding mode and key residues involved in this interaction (see sample link placeholder: https://golgi.sandbox.google.com/). The resulting docked complexes were visualized and further analyzed using PyMOL molecular graphics software (http://pymol.sourceforge.net/).

### Statistical analysis

All statistical analyses were performed using GraphPad Prism 8.0 (GraphPad Software) or SPSS 25.0 (IBM). Data are presented as the mean ± standard deviation (SD) from at least three independent biological replicates. The normality of data distribution was assessed using the Shapiro-Wilk test, and the homogeneity of variances was confirmed with Levene’s test (or *F*-test, as appropriate). For comparisons between two groups of parametric data, Student’s *t*-test was used; one-way ANOVA was applied for multi-group comparisons. For non-parametric data, the Mann-Whitney U test (two groups) or the Kruskal-Wallis test (multiple groups) was employed. Survival curves were generated by the Kaplan-Meier method and compared using the log-rank test. Correlations were analyzed using Pearson’s coefficient for normally distributed data and Spearman’s coefficient for non-normally distributed data. Sample sizes were determined by power analysis (*α* = 0.05, power = 0.80) to reliably detect moderate effect sizes. Statistical significance was set at *p* < 0.05 (two-tailed) for all tests unless otherwise stated.

## Results

### Reduced MAGI3 expression correlates with poor prognosis in ccRCC

**Fig. 1:**
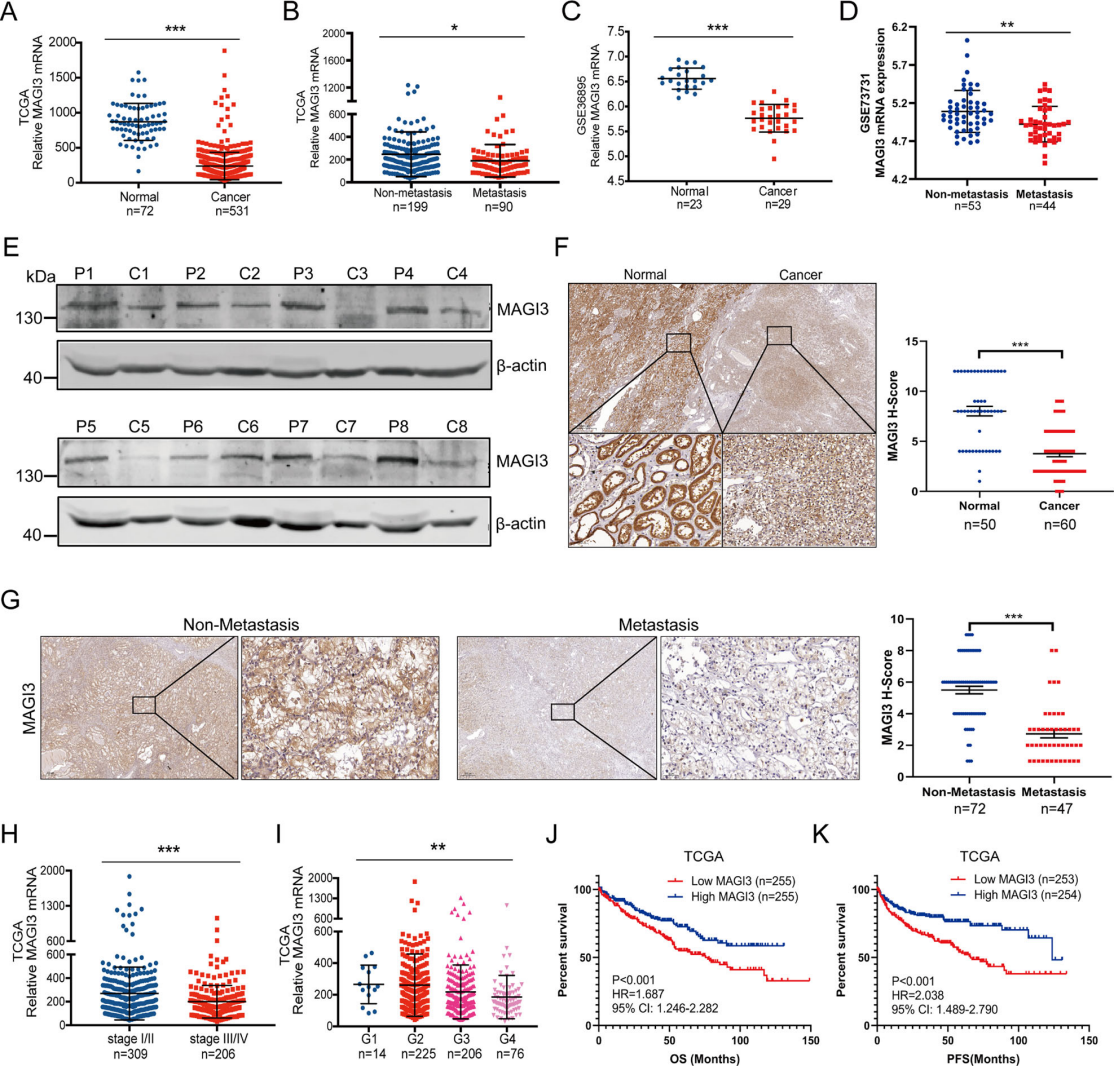
Reduced MAGI3 expression correlates with metastasis and poorer prognosis in ccRCC, indicating it as a potential protective factor. **A** Scatter plots showing the relative MAGI3 mRNA expression in ccRCC and adjacent normal tissues. **B** Scatter plots showing the relative MAGI3 mRNA expression in ccRCC patients with or without metastasis. **C**, **D** Scatter plots showing the relative MAGI3 mRNA expression in ccRCC and adjacent normal tissues from GSE36895, ccRCC patients with or without metastasis from GSE73731 datasets. *p < 0.05, **p < 0.01, ***p < 0.001, determined by an independent sample *t*-test. **E** Western blot analysis reveals reduced MAGI3 protein levels in human ccRCC. P: paracancerous tissue; C: cancer tissue. **F** Immunohistochemical staining (IHC) showing low expression of MAGI3 in ccRCC compared to adjacent normal tissue. Scale bars, 500 μm (overview) and 50 μm (zoomed). ***p < 0.001, independent sample *t*-test (right panel). **G** Lower level of MAGI3 protein is associated with ccRCC metastasis. Representative images of IHC staining of MAGI3 in non-metastatic or metastatic ccRCC specimens. Scale bars: 500 μm (overview) and 50 μm (zoomed, left panel). Downregulation of MAGI3 mRNA levels in advanced stages (**H**) or high grades (**I**) of ccRCC tissues, based on TCGA data. (**p < 0.01, ***p < 0.001). **J**, **K** Correlation of low MAGI3 expression with poor survival outcomes in ccRCC. Kaplan-Meier Survival Analysis comparing OS or PFS in TCGA ccRCC cohorts dichotomized based on the median value of MAGI3 mRNA expression.

To identify key molecular drivers of ccRCC progression, we focused on the role of metastasis, which is a major contributor to patient mortality [29]. We performed differentially expressed genes (DEGs) analysis between ccRCC tissues and adjacent normal tissues (GSE12606) as well as between metastatic ccRCC tissues and non-metastatic ccRCC samples (GSE31232). Using GEO2R analysis, we identified 1827 DEGs in primary ccRCC and 40 DEGs specifically associated with metastasis (Fig. S1A). Intersection analysis revealed five overlapping downregulated genes: MAGI3, EGF, ADH1C, EYA4, and C3orf52 (Fig. S1B). Further analysis of the TCGA-KIRC cohort revealed that EGF, ADH1C, EYA4, and C3orf52 were downregulated in tumor tissues (Fig. S2A) but unchanged in metastases (Fig. S2B). Strikingly, MAGI3 exhibited progressive suppression: significantly reduced in primary tumors (Fig. 1A) and further downregulated at metastatic sites (Fig. 1B). These downregulation was consistently observed across multiple independent datasets (Fig. 1C, D).

Protein-level validation confirmed these findings: Western blotting revealed diminished MAGI3 in ccRCC versus adjacent tissues (Fig. 1E), while immunohistochemistry demonstrated maximal suppression in cancer tissues and metastatic lesions (Fig. 1F, G).

Clinically, diminished MAGI3 expression correlated with aggressive disease features including advanced stage (Fig. 1H, Fig. S3A), higher Fuhrman grade (Fig. 1I, Fig. S3B), and inferior overall survival (OS) and progression-free survival (PFS) (Fig. 1J, K). Critically, these associations proved independent of VHL mutation status (Fig. S3C). Multivariate Cox regression confirmed MAGI3 as an independent prognostic factor for OS (Supplementary Tables 1).

To validate these findings in an independent metastatic cohort, we analyzed MAGI3 expression in lung metastases from ccRCC patients (GSE22541 cohort, *n* = 41). Patients with high metastatic burden (8 or more nodules) showed significantly lower MAGI3 expression compared to those with low burden (6 or fewer nodules, Fig. S3D). Similarly, patients who experienced early disease recurrence (DFI ≤ 15 months) showed reduced MAGI3 expression relative to those with longer disease-free intervals (>30 months) (Fig. S3E). Critically, stratification by MAGI3 expression in metastatic lesions revealed markedly shorter progression-free survival in the low-expression cohort (Fig. S3F).

Together, these results highlight MAGI3 as a clinically robust prognostic marker in ccRCC, relevant in both primary tumors and metastatic sites.

### MAGI3 inhibits ccRCC cell migration, invasion and metastasis

**Fig. 2:**
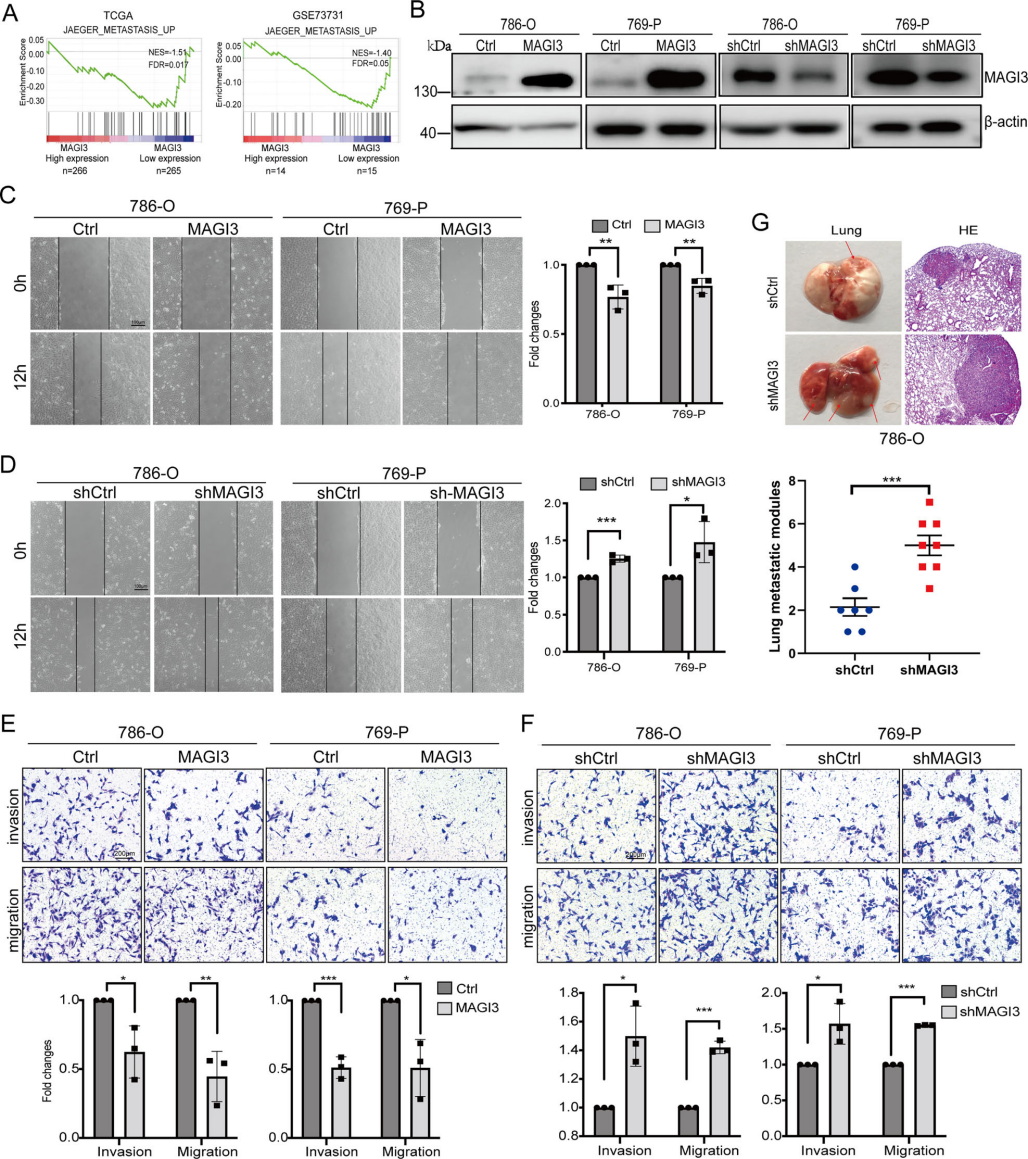
MAGI3 suppresses the migration, invasion, and metastasis in ccRCC cells. **A** Analysis of metastasis-related upregulated gene sets enriched in ccRCC specimens with low MAGI3 expression. Gene expression data from TCGA or GSE73731 were subjected to GSEA to identify pathways associated with metastasis. **B** Validation of stable MAGI3 overexpression or knockdown in 786-O and 769-P cells using western blotting. **C** Ectopic expression of MAGI3 suppresses migration in 786-O and 769-P cells. **D** Knockdown of MAGI3 promotes migration in 786-O and 769-P cells, demonstrated by in vitro scratch assay. **E** Overexpression of MAGI3 inhibits migration and invasion in 786-O and 769 cells. **F** Knockdown of MAGI3 promotes migration and invasion in 786-O and 769 cells, assessed by transwell assays. **G** Knockdown of MAGI3 expression promotes lung metastasis in a ccRCC xenograft model. 786-O/shCtrl or 786-O/shMAGI3 cells were injected into SCID/NOD mice (*n* = 15) via tail vein. The mice were sacrificed after 3 months, and the number of pulmonary metastatic nodules was statistically analyzed.

The consistent downregulation of MAGI3 in metastatic ccRCC strongly suggested its functional role in tumor progression. To explore this possibility, we performed GSEA on TCGA-KIRC and GSE73731 datasets. This analysis revealed significant enrichment of metastasis-associated gene signatures in tumors with low MAGI3 expression (Fig. 2A), suggesting its potential involvement in metastatic processes.

To functionally characterize MAGI3 in ccRCC, we modulated its expression in 786-O and 769-P cell lines. We generated stable MAGI3-overexpressing cell lines and performed MAGI3 knockdown using lentiviral shRNA transduction (Fig. 2B) following validation of knockdown efficiency (Fig. S4) [28]. Functional assays revealed that MAGI3 overexpression significantly impaired both migration and invasion capabilities in transwell and wound healing assays (Fig. 2C, E). Conversely, MAGI3 knockdown markedly enhanced these metastatic properties (Fig. 2D, F). Most compellingly, in vivo experiments showed that injection of MAGI3-knockdown 786-O cells resulted in a significantly greater number of lung metastatic foci compared to controls (Fig. 2G). Collectively, these robust findings establish MAGI3 as a potent suppressor of ccRCC cell metastasis.

### MAGI3 promotes β-catenin degradation through the ubiquitin-proteasome pathway

**Fig. 3:**
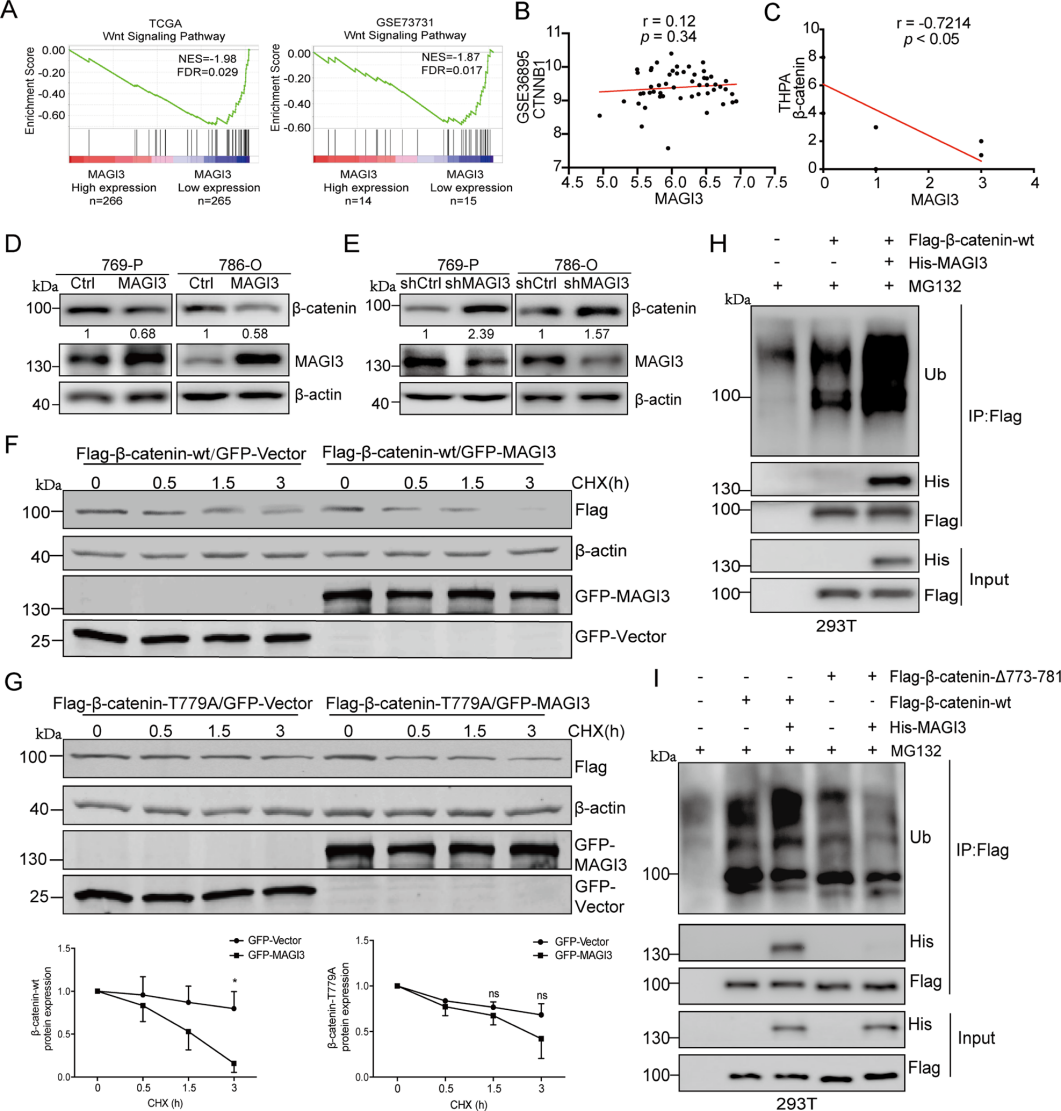
MAGI3 mediates β-catenin protein degradation via ubiquitination. **A**–**C** MAGI3 exhibits a negative correlation with β-catenin protein level and Wnt signaling activation in ccRCC specimens. Low MAGI3 level was associated with Wnt signaling activation in ccRCC, as evidenced by enriched gene signatures of Wnt/β-catenin activation in patients with lower MAGI3 levels in TCGA KIRC and GSE73731 datasets (**A**). MAGI3 does not correlate with β-catenin at mRNA levels in clinical specimens (**B**). MAGI3 negatively correlates with β-catenin at protein levels in THPA KIRC datasets (**C**). **D**, **E** Overexpression of MAGI3 reduces β-catenin protein level, while knockdown of MAGI3 increased β-catenin protein level in 786-O or 769-P cells. Cells stably transfected with MAGI3 or Ctrl (**D**), or knockdown with shMAGI3 or shCtrl constructs (**E**), were subjected to western blotting analysis to measure the levels of β-catenin, MAGI3 and β-actin protein. **F**, **G** Overexpression of MAGI3 decreased the half-lives of β-catenin protein via its interaction with MAGI3. 293T cells were transiently transfected with Flag-β-catenin-wt (**F**) or Flag-β-catenin-T779A (**G**) in absence or presence of GFP-MAGI3 respectively, were treated with CHX (25 μg/mL) for the indicated time before cell harvest for western blotting. Band density was quantified using ImageJ software. Data represent three independent experiments (*n* = 3). *p < 0.05; ns, no significant difference (*t* test). **H** Overexpression of MAGI3 promotes β-catenin ubiquitination. **I** The interaction with β-catenin is essential for MAGI3 to promote β-catenin ubiquitination. 293T cells were transiently transfected with Flag-β-catenin-wt or Flag-β-catenin-Δ773-781 in absence or presence of His-MAGI3 respectively, were treated with MG132 for 10 h. Cell lysates were subjected to IP with anti-Flag antibody-coupled beads. The precipitated complexes were probed with anti-ubiquitin antibody to detect ubiquitinated Flag-β-catenin.

To determine how MAGI3 exerts its anti-metastatic effects, we performed systematic pathway enrichment analyses. Notably, GSEA identified Wnt/β-catenin signaling as the most negatively correlated pathway with MAGI3 expression in both TCGA-KIRC and GSE73731 datasets (Fig. S5A, Fig. 3A). Given this strong link to Wnt signaling, we used UALCAN and DAVID to validate MAGI3’s role in this pathway. These analyses further verified that MAGI3 associated with protein phosphorylation and ubiquitin-mediated degradation pathways (Fig. S5B).

Although MAGI3 and β-catenin mRNA levels were not significantly correlated in clinical samples (Fig. 3B), we observed a robust inverse correlation at the protein level (Fig. 3C). To determine whether this relationship was causative, we modulated MAGI3 expression in ccRCC cells. Consistent with our clinical observations, MAGI3 overexpression reduced β-catenin protein levels (Fig. 3D), whereas MAGI3 knockdown increased them (Fig. 3E). Given the well-established regulation of β-catenin stability via the ubiquitin-proteasome system [29], we next investigated whether MAGI3 influences β-catenin turnover. Notably, MAGI3 overexpression significantly accelerated the degradation of wild-type β-catenin (Fig. 3F). In contrast, MAGI3 had no effect on the stability of a β-catenin-T779A mutant (Fig. 3G), which disrupts the canonical PDZ-binding motif required for MAGI3 binding [30]. We therefore hypothesized that MAGI3 promotes β-catenin ubiquitination. Indeed, MAGI3 enhanced the ubiquitination of wild-type β-catenin (Fig. 3H). Critically, this enhancement was completely abolished upon deletion of the C-terminal PDZ-binding motif (β-catenin-Δ773-781 mutant; Fig. 3I). These data collectively demonstrate that the physical interaction between MAGI3 and the C-terminus of β-catenin is a prerequisite for MAGI3-mediated ubiquitination and subsequent proteasomal degradation of β-catenin.

### MAGI3 regulates β-catenin phosphorylation and ubiquitination via C-tail interactions

**Fig. 4:**
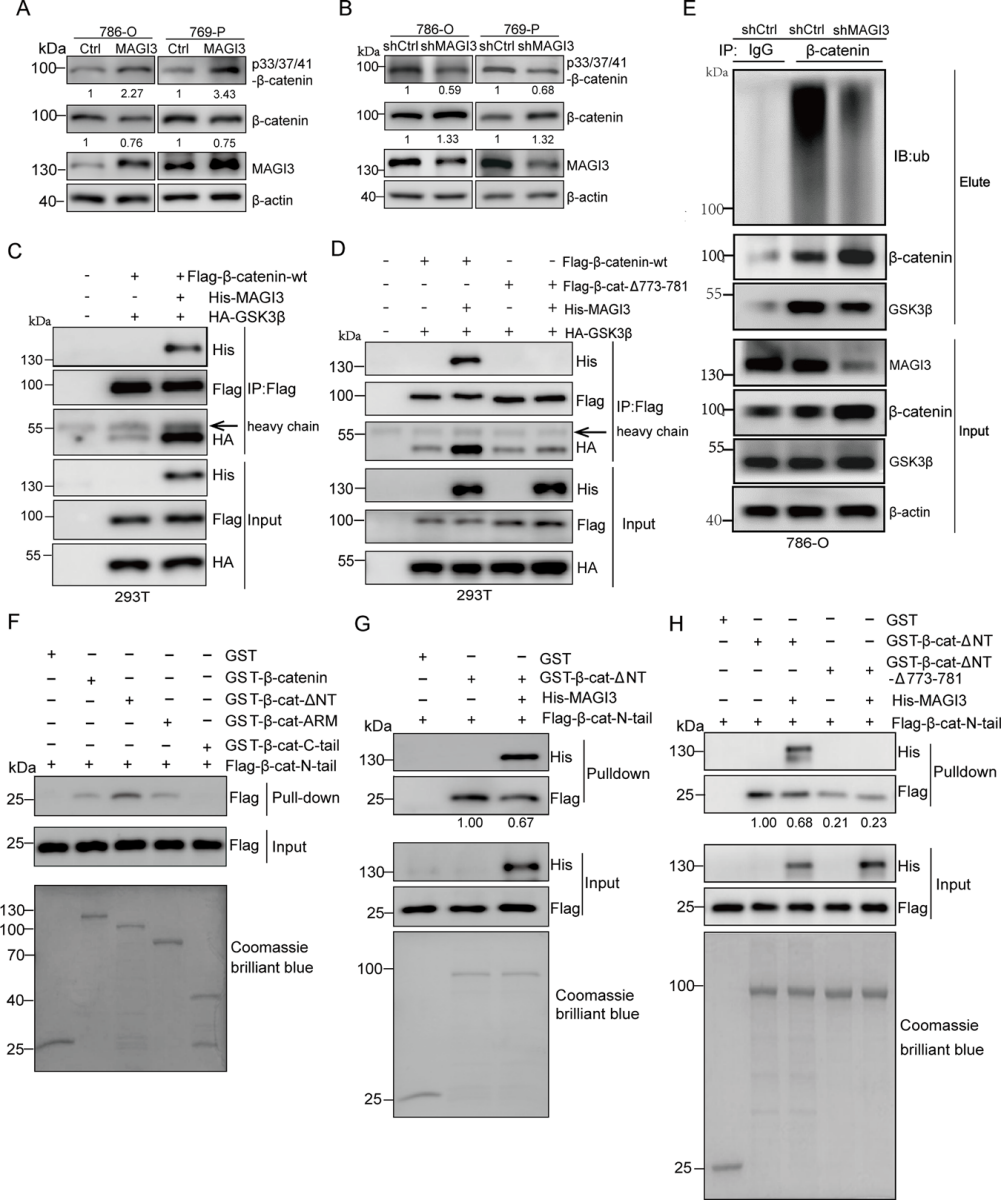
MAGI3 facilitates β-catenin phosphorylation and degradation by enhancing GSK3β association and disrupting N-tail intramolecular interactions. **A**, **B** Overexpression MAGI3 enhanced β-catenin phosphorylation and reduces β-catenin protein level in 786-O or 769-P cells. Knockdown MAGI3 reduces β-catenin phosphorylation and increases β-catenin protein level in 786-O or 769-P cells. Cell stably transfected with MAGI3 or Ctrl (**A**), or knockdown with shMAGI3 or shCtrl (**B**) constructs respectively, were subjected to western blotting analysis. Phosphorylation of β-catenin at Ser33/Ser37/Thr41 was detected with anti-phospho-β-catenin (Ser33/Ser37/Thr41) antibody. **C** MAGI3 serves as a scaffold protein to facilitate the interaction between GSK3β and β-catenin. **D** The C-terminus of β-catenin plays an indispensable role in MAGI3-mediated enhancement of the association between GSK3β and β-catenin. 293T cells were transiently transfected with Flag-β-catenin-wt or Flag-β-catenin-Δ773-781 (Flag-β-cat-Δ773-781) along with HA-GSK3β in presence or absence of His-MAGI3. Lysates were precipitated with an anti-Flag antibody and blotted with anti-His, anti-Flag or anti-HA antibody respectively. **E** Knockdown of MAGI3 inhibits the endogenous interaction between GSK3β and β-catenin, and suppresses the ubiquitination of β-catenin. Cell lysates were subjected to IP with anti-β-catenin antibody-coupled beads. The precipitated complexes were probed with anti-β-catenin, anti-ubiquitin and anti-GSK3β antibody to detect ubiquitinated β-catenin and its interaction with GSK3β. **F** Intramolecular interaction of β-catenin N-tail (β-cat-N-tail) with its ARM (β-cat-ARM) domain forms a loop structure. β-catenin GST fusion proteins containing the indicated domains were utilized to pull down cell lysates from 293T cells transfected with Flag-β-cat-N-tail, and the pull downed complexes were detected by western blotting. **G** MAGI3 significantly reduces the binding of extra molecular Flag-β-cat-N-tail with β-catenin. **H** The C-terminus of β-catenin was indispensable for MAGI3-mediated reduction in the interaction of extra molecular Flag-β-cat-N-tail with β-catenin. β-catenin GST fusion proteins containing the indicated domains were used to pull down cell lysates from 293T transfected with Flag-β-cat-N-tail with or without His-MAGI3, and the pull downed complexes were detected by western blotting.

We next examined whether MAGI3 influences glycogen synthase kinase 3β (GSK3β)-dependent phosphorylation of β-catenin at Ser33/Ser37/Thr41 residues—critical sites triggering ubiquitination and degradation [30]. Strikingly, MAGI3 overexpression substantially enhanced phosphorylation at these residues (Fig. 4A), while MAGI3 knockdown reduced phosphorylation by ~60% relative to scrambled shRNA controls (Fig. 4B). This establishes MAGI3 as a scaffold facilitating GSK3β-mediated β-catenin phosphorylation. Co-immunoprecipitation in 293T cells transfected with Flag-β-catenin, His-MAGI3, and HA-GSK3β confirmed MAGI3 strengthens β-catenin–GSK3β complex assembly (Fig. 4C). Critically, this enhancement was abolished when using β-catenin-Δ773-781—a C-terminal deletion mutant lacking the PDZ-binding motif essential for MAGI3 interaction (Fig. 4D). These findings indicate that complex formation is driven by direct engagement at the C-terminus. In 786-O ccRCC cells, MAGI3 depletion disrupted endogenous β-catenin–GSK3β interactions and impaired β-catenin polyubiquitination (Fig. 4E). Taken together, these data demonstrate MAGI3 binding to β-catenin’s C-terminal PDZ binding motif enables GSK3β-mediated N-terminal phosphorylation and subsequent degradation.

β-catenin comprises three functional domains: N-terminal (N-tail), Armadillo repeats (ARM), and C-terminal (C-tail). While GSK3β phosphorylates N-tail residues, MAGI3 binds the C-tail PDZ motif (D-T-D-L) via its PDZ4 domain [31]. GST pull-down assays with purified β-catenin domains (Fig. S6A) revealed β-catenin’s intrinsic N-tail–ARM association (Fig. 4F, *lane 4*), which stabilized with an intact C-tail (Fig. 4F, *lane 3*). MAGI3 binding disrupted this intramolecular interaction (Fig. 4G). Conversely, β-catenin lacking the terminal 8 C-tail residues exhibited weakened N-tail–ARM binding independent of MAGI3 (Fig. 4H), confirming the C-tail maintains structural stability.

**Fig. 5:**
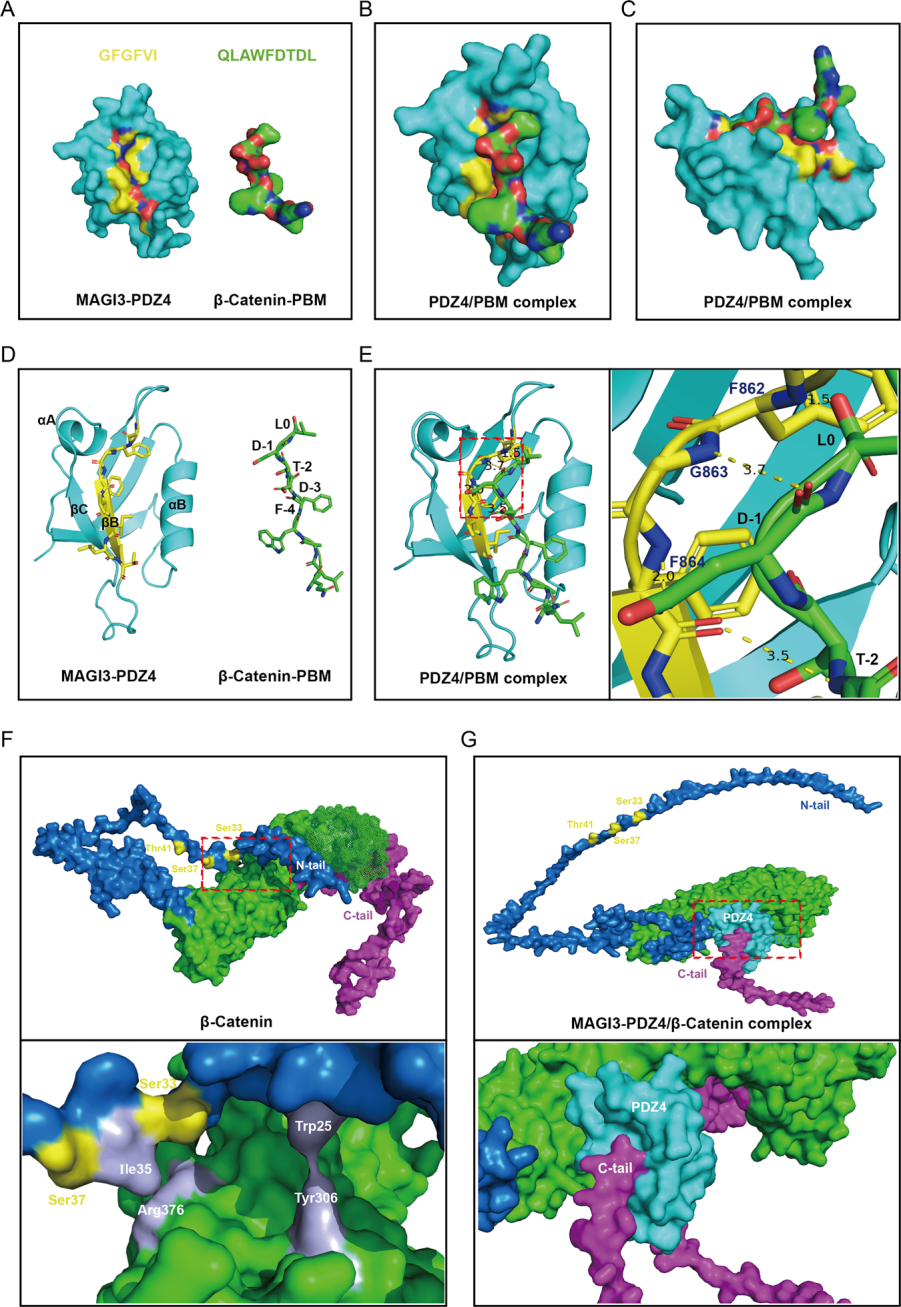
Molecular interaction between the β-catenin C-terminal tail and the MAGI3-PDZ4 domain and its functional consequence on β-catenin conformation. **A** Structure of the MAGI3-PDZ4 domain (gray) predicted by AlphaFold, highlighting the carboxylate-binding loop (yellow, residues 861-866). The bound peptide corresponding to the β-catenin PDZ-binding motif (PBM, green, residues 773-781) is shown with oxygen (red) and nitrogen (blue) atoms. Front (**B**) and side (**C**) views of the simulated binding interface between MAGI3-PDZ4 (gray) and the β-catenin PBM (green), illustrating the spatial constraints of the interaction. **D** Ribbon diagram of the MAGI3-PDZ4:β-catenin-PBM complex. Coloring is consistent with (**A**). **E** Detailed stereo view of the hydrogen-bonding network (dashed lines) at the binding interface. Magnified views of the boxed regions show key atomic interactions within the wireframe models. **F** Schematic of the β-catenin domain architecture, depicting ARM repeats (green), the N-terminal tail (N-tail, blue), the C-terminal tail (C-tail, purple), and the locations of key GSK3β phosphorylation sites (yellow stars). **G** Working model proposing that binding of MAGI3-PDZ4 to the β-catenin C-tail prevents the intramolecular interaction between the C-tail and the N-tail, thereby potentially exposing the N-terminal phosphorylation sites.

AlphaFold simulations corroborated MAGI3’s PDZ4 domain engages β-catenin’s PDZ-binding motif through hydrophobic contacts (Fig. 5A–E). Structural analysis showed β-catenin folds into a toroidal architecture, with the N-tail forming a hydrogen bond (Trp25/Ile35–Tyr306/Arg376) to the ARM domain (Fig. 5F). We proposed that binding of MAGI3 to the C-terminal tail of β-catenin disrupts this N-tail–ARM interaction, thereby exposing the N-terminal phosphodegron (Fig. 5G). To test this model experimentally, we performed FLIM-FRET analysis. The results showed that MAGI3 co-expression significantly increased the average fluorescence lifetime between CFP-β-cat-N-tail and YFP-β-cat-ΔNT (Fig. S6B), indicating a disruption of their intramolecular interaction. Collectively, these data provide direct experimental evidence that MAGI3 binding to the C-terminal PBM allosterically inhibits the intramolecular interaction between the N-tail and the ARM domain of β-catenin (Fig. S6C).

### MAGI3 inhibits ccRCC metastasis and rapalog resistance by suppressing β-catenin signaling

**Fig. 6:**
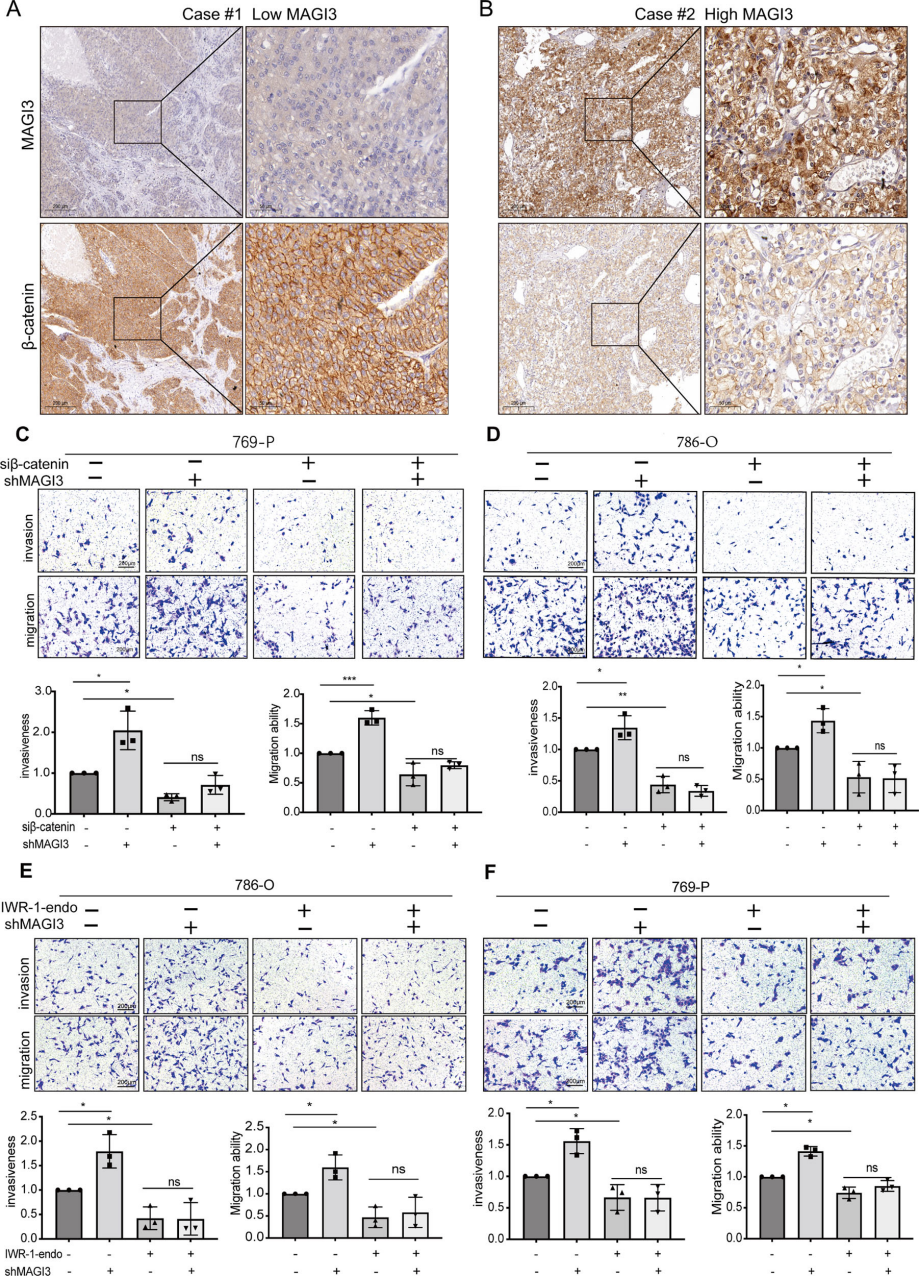
MAGI3 suppresses ccRCC cell migration and invasion through inhibition of Wnt/β-catenin signaling. **A**, **B** IHC analysis of MAGI3 and β-catenin expression in ccRCC specimens from 119 patients. Representative images of MAGI3 and β-catenin staining in patients with low and high MAGI3 expression levels. Scale bars: 200 μm. Magnified views of the wireframe regions are shown in the right panels. Scale bars: 50 μm. **C**, **D** Knockdown of β-catenin rescues the increased cell migration and invasion induced by MAGI3 knockdown. 786-O and 769-P cells were transfected with shMAGI3 alone, or in combination with β-catenin siRNA. Migration and invasion were assessed using transwell assays. **E**, **F** Blocking Wnt/β-catenin signaling rescues the increased cell migration and invasion induced by MAGI3 knockdown. 786-O and 769-P cells were transfected with shMAGI3 alone, or in combination with IWR-1-endo, an inhibitor of the Wnt/β-catenin pathway. Migration and invasion were assessed using transwell assays (*t* test; *p < 0.05; **p < 0.01; ***p < 0.001; ns, no significant difference; values represent mean ± SD, *n* = 3).

To substantiate MAGI3’s role in β-catenin degradation, we analyzed clinical ccRCC specimens. Immunohistochemistry revealed significantly elevated β-catenin protein levels in tumors with low MAGI3 expression (Fig. 6A), contrasting with robust β-catenin reduction in MAGI3-high specimens (Fig. 6B). This inverse correlation was statistically validated (Fig. S7A). GSEA of renal cancer lung metastases (GSE22541) further demonstrated pronounced activation of canonical WNT/β-catenin signaling in patients exhibiting poor prognosis and low MAGI3 expression (Fig. S7B, C).

Consistent with this inverse correlation, depletion of MAGI3 not only promoted the accumulation of β-catenin (Fig. S7D, E, *lanes 1–2*) but also significantly enhanced the invasive and migratory capacities of cancer cells (Fig. 6C, D*lanes 1–2*). Importantly, these MAGI3 deficiency-driven phenotypes were effectively rescued by either genetic knockdown of β-catenin (Fig. 6C, D; Fig. S7D, E, *lanes 3–4*) or pharmacological inhibition of the Wnt pathway using IWR-1-endo (Fig. 6E, F). These results establish that the anti-metastatic function of MAGI3 is primarily mediated through its suppression of β-catenin. To further corroborate the impact of MAGI3 on Wnt/β-catenin transcriptional activity, we analyzed the expression of key target genes (CCND1, SNAI1, CD44) by qPCR. MAGI3 overexpression significantly suppressed, whereas its knockdown markedly upregulated, the transcription of these genes (Fig. S7F, G). Furthermore, immunofluorescence analysis demonstrated that MAGI3 and β-catenin co-localize primarily in the cytoplasmic compartment (Fig. S8), providing spatial context for their functional interaction. Collectively, these data demonstrate that MAGI3 acts as a bidirectional modulator of the oncogenic Wnt/β-catenin-driven gene program.

**Fig. 7:**
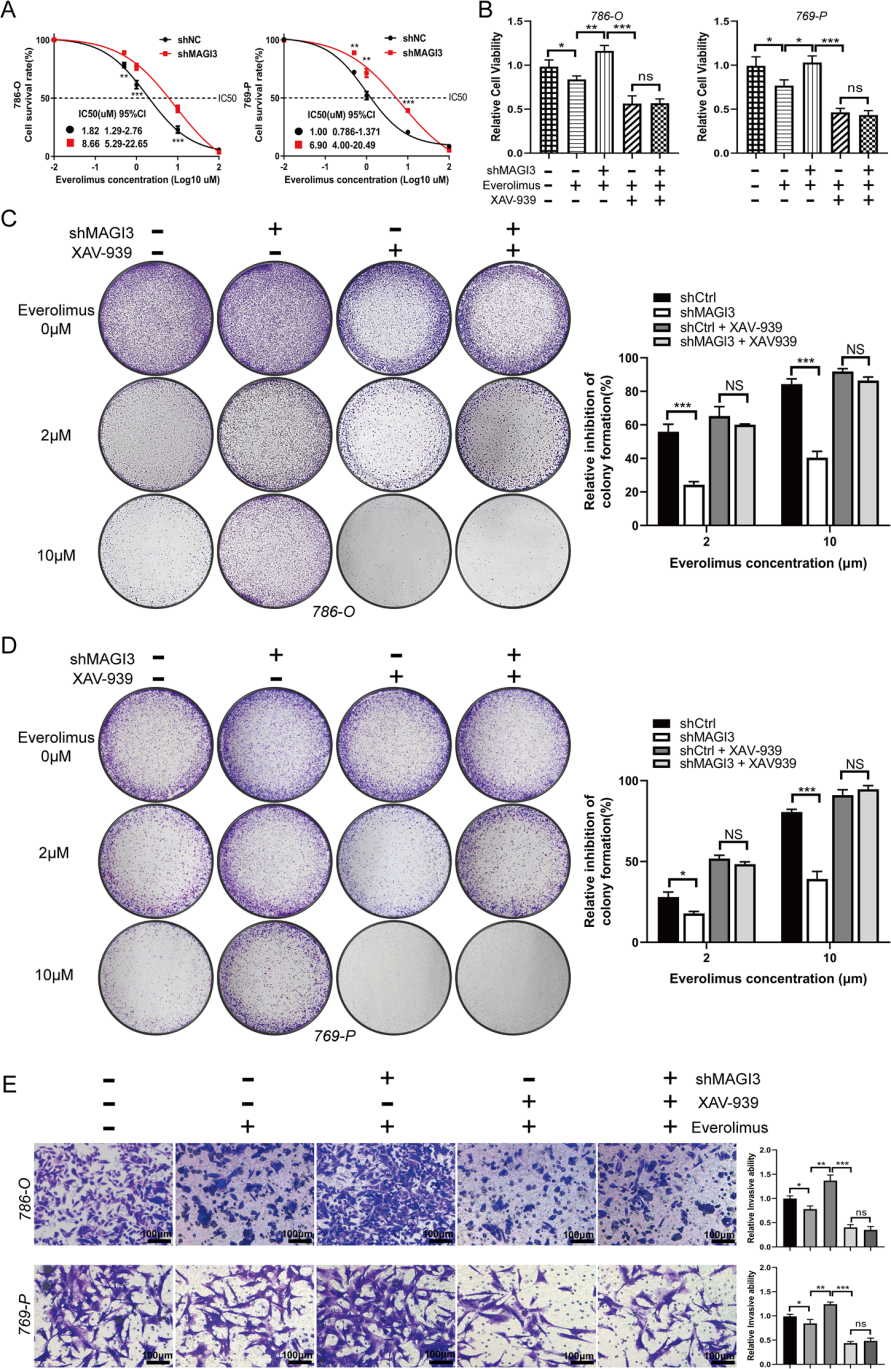
MAGI3 enhances sensitivity to rapalogs in ccRCC by suppressing Wnt/β-catenin signaling. **A** Knockdown of MAGI3 increased the IC_50_ of Everolimus in 786-O and 769-P cells. Dose-response survival curves of MAGI3-knockdown 786-O and 769-P cells exposed to increasing concentrations of Everolimus for 48 h. Mean ± SD; *n* = 3; two-way ANOVA. **B** The reduced drug sensitivity caused by MAGI3 knockdown is restored by treatment with XAV-939, an inhibitor of the Wnt/β-catenin pathway. Cell viability of 786-O and 769-P cells was determined using CCK-8 assays. **C**, **D** MAGI3 knockdown promotes clonogenic formation in Everolimus-treated 786-O and 769-P cells, while concomitant treatment with XAV-939 and Everolimus markedly inhibits clonogenic formation. Colony formation assays were conducted in 786-O and 769-P cells treated with Everolimus in the absence or presence of XAV-939 for 14 days. Quantification analysis of clone formation assays. **E** Combination treatment with XAV939 and Everolimus synergistically inhibits cell invasion of ccRCC. Transwell invasion assays were conducted in 786-O and 769-P cells treated with Everolimus in the absence or presence of XAV-939 for 24 h. (*t* test; *p < 0.05; **p < 0.01; ***p < 0.001; ns, no significant difference; values represent mean ± SD, *n* = 3).

Given the established crosstalk between Wnt/β-catenin and PI3K/Akt/mTOR pathways in ccRCC therapeutics [24, 32, 33], we evaluated MAGI3’s role in modulating rapalog sensitivity. MAGI3 knockdown in 786-O and 769-P cells significantly elevated Everolimus IC_50_ values (Fig. 7A), indicating induced drug resistance. This phenotype was reversed by co-administration of the Wnt inhibitor XAV-939 (Fig. 7B). Colony formation assays confirmed MAGI3 loss diminished Everolimus responsiveness, while XAV-939/Everolimus combination therapy potently suppressed clonogenicity (Fig. 7C, D). Parallel invasion assays demonstrated synergistic suppression of metastatic potential (Fig. 7E), indicating dual-pathway blockade overcomes compensatory resistance mechanisms.

**Fig. 8:**
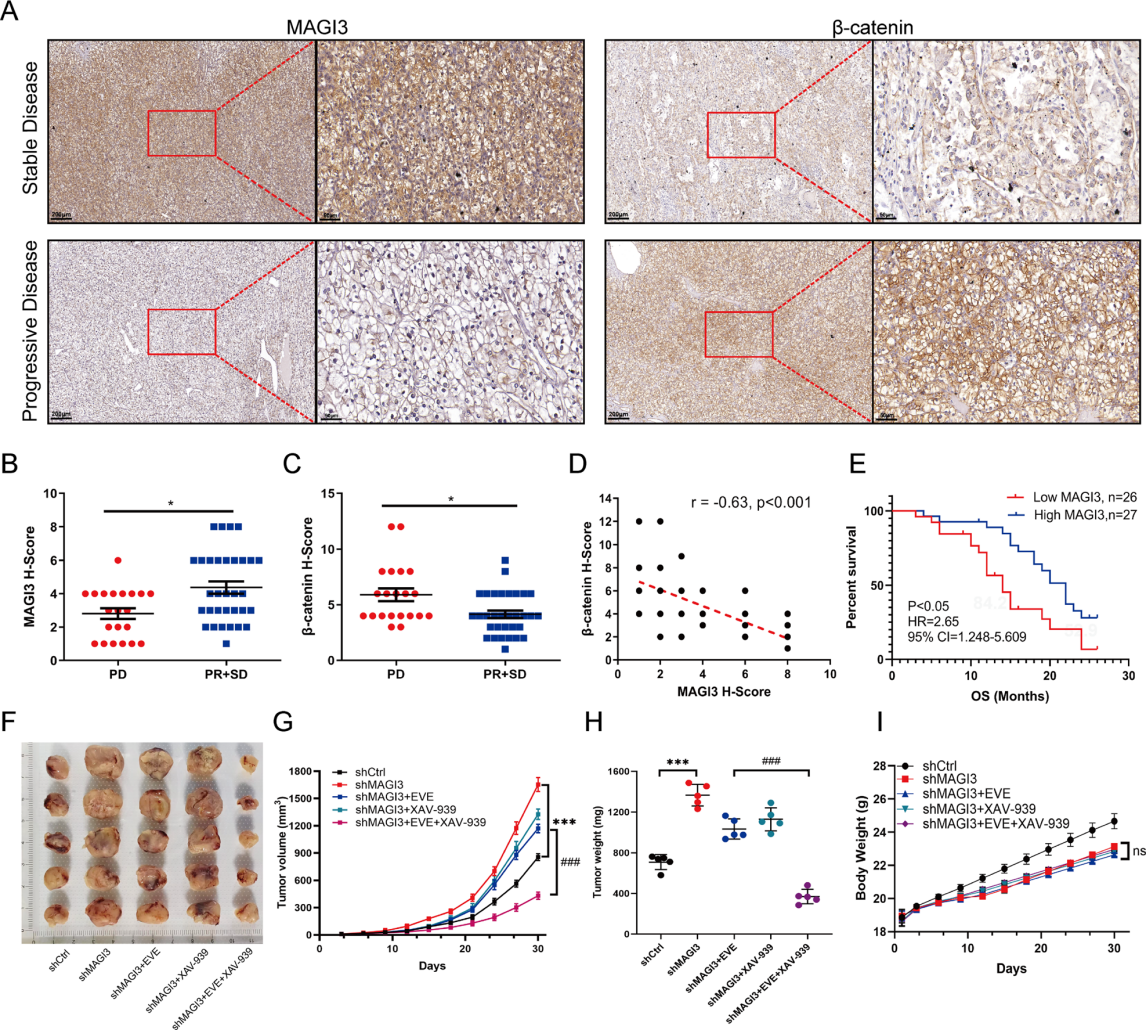
Association of MAGI3 and β-catenin expression with therapeutic response and survival in everolimus-treated ccRCC patients, and efficacy of combination therapy in MAGI3-deficient xenografts. **A** Representative immunohistochemical staining of MAGI3 and β-catenin in tumor tissues from 53 ccRCC patients treated with everolimus. Sections from responders (stable disease or partial response) and non-responders (progressive disease) are shown. Left: overview (scale bar, 200 μm); right: higher-magnification views of the boxed areas (scale bar, 50 μm). H-score analysis of MAGI3 (**B**) and β-catenin (**C**) expression in tumors from responders and non-responders. Data are presented as mean ± SD. Differences between groups were assessed by the Mann-Whitney *U* test (*P < 0.05, **P < 0.01). **D** Scatter plot showing a significant negative correlation between MAGI3 and β-catenin expression levels (Spearman’s *r* = –0.63, *P* < 0.001). **E** Kaplan-Meier overall survival analysis of patients stratified by high or low MAGI3 expression. Statistical significance was determined by the log-rank test. **F**–**I** In vivo efficacy and safety of combined mTOR and Wnt inhibition. **F** Representative images of tumors resected from each group at the end of the study. **G** Tumor growth curves of mice bearing shMAGI3 xenografts (*n* = 5 per group) treated with vehicle, Everolimus (EVE, 5 mg/kg), XAV-939 (XAV, 10 mg/kg), or the combination (EVE + XAV). The shCtrl group is shown as a reference. **H** Final tumor weights for each group. **I** Body weight changes of mice during the treatment period, indicating treatment tolerability. Data in **G**–**I** are shown as mean ± SD. Statistical significance was determined by two-way ANOVA (**G**, **I**) or one-way ANOVA (**H**) followed by post-hoc tests (***P < 0.001, shCtrl vs. shMAGI3; ###*P* < 0.001, combination therapy vs. shMAGI3+EVE monotherapy; ns not significant).

Analysis of 53 Everolimus-treated ccRCC patient samples revealed distinct expression patterns: responding patients (SD/PR) exhibited high MAGI3 with low β-catenin, whereas PD correlated with inverse profiles (Fig. 8A–C). Immunohistochemistry quantified a strong negative MAGI3-β-catenin correlation (*r* = −0.63; Fig. 8D). Critically, low MAGI3 expression predicted significantly reduced overall survival (Fig. 8E), positioning MAGI3 as both a mechanistic regulator and clinical biomarker for rapalog response.

To functionally validate the association between MAGI3 loss and therapeutic resistance in patients, and to explore a corresponding treatment strategy, we employed a subcutaneous xenograft model. To this end, mice bearing tumors derived from MAGI3-knockdown (shMAGI3) 786-O cells, which recapitulate the aggressive growth phenotype (Fig. 8F), were randomized into four treatment groups: vehicle control, Everolimus (EVE), XAV–939, and EVE + XAV–939. Consistent with patient data, shMAGI3 tumors were largely resistant to EVE monotherapy. XAV–939 alone also had only a modest effect. However, the combination of EVE and XAV-939 achieved a marked and synergistic suppression of tumor growth, yielding final tumor volumes and weights that were significantly lower than those in all other groups (Fig. 8G, H). Importantly, the potent anti-tumor efficacy of the combination therapy was achieved without inducing significant systemic toxicity. Mice in all treatment groups maintained stable body weights throughout the study period (Fig. 8I), and no treatment-related adverse events or mortality were observed. Collectively, these in vivo data demonstrate that dual-pathway inhibition synergistically overcomes MAGI3 deficiency-driven tumor growth and resistance, providing a compelling rationale for co-targeting mTOR and Wnt/β-catenin in this aggressive ccRCC subtype.

## Discussion

Despite therapeutic advances, metastatic clear cell renal cell carcinoma (mccRCC) maintains a dismal 5-year survival rate below 15%. This underscores an urgent need for biomarkers capable of stratifying aggressive disease. Through multi-cohort analysis, we identify MAGI3 transcriptional silencing as a hallmark of lethal progression. Mechanistically, MAGI3 depletion correlates with escalating metastatic burden—from initial tumorigenesis (Fig. 1A, C) to distant colonization (Fig. 1B, D, G)—and independently elevates mortality risk regardless of VHL status (Supplementary Tables 1). These findings position MAGI3 loss not merely as a correlative marker, but as an active driver of metastatic competence.

Of particular interest, the transcriptional downregulation of MAGI3 shares key molecular features with chronic inflammatory disorders. Building on this conserved regulatory axis, multiple pathogenically active miRNAs (miR-20b-5p, miR-34c-3p, miR-5692) identified in inflammatory bowel disease [34–36] directly target MAGI3—mirroring inflammation-driven miRNA networks operational in ccRCC tumorigenesis [37, 38]. While epigenetic lesions or somatic mutations may coexist with this phenomenon, the perpetual inflammatory milieu characteristic of ccRCC microenvironments strongly implicates stroma-derived repressive signaling as the dominant regulatory layer. Nevertheless, future work should clarify whether inflammatory signaling or specific miRNAs directly orchestrate MAGI3 loss in ccRCC.

Our functional studies in ccRCC cells revealed that MAGI3 expression inhibited cell migration and invasion by both wound healing and transwell assays (Fig. 2C–F). Our in vivo lung metastasis model using MAGI3-knockdown cells further supported the inhibitory role of MAGI3 in ccRCC progression (Fig. 2G), these findings highlight MAGI3 as a key suppressor of metastatic behavior in ccRCC. It is worth noting that while our subcutaneous xenograft model demonstrates MAGI3’s role in restraining primary tumor growth (Fig. 8F–H, shCtrl vs. shMAGI3)—a prerequisite for metastasis—the specific step(s) of the metastatic cascade (e.g., intravasation, circulating tumor cell survival, or distant colonization) that are most critically regulated by MAGI3 merit further dissection in future studies using extended-duration tail-vein assays or other organotropic metastasis models. Additionally, we established a connection between MAGI3 dysregulation and the activation of the Wnt/β-catenin pathway, a well-established driver of ccRCC progression. Knockdown of MAGI3 not only activated the Wnt/β-catenin pathway by upregulating β-catenin at the protein level (Fig. 4B and Fig. S7D, E) but also facilitated ccRCC cell invasion and migration (Fig. 6C, D). Our rescue experiments demonstrated that both β-catenin knockdown and pharmacological inhibition of the Wnt/β-catenin pathway could counteract the pro-invasive and migratory effects induced by MAGI3 knockdown (Fig. 6C–F).

Building on our earlier work identifying MAGI3 as a negative regulator of Wnt/β-catenin signaling in glioma and cervical cancer models [31, 39], where its expression consistently correlated with reduced tumor aggressiveness, MAGI3 emerged as a multi-cancer tumor suppressor. Yet its precise mechanism remained unclear. Our current data now reveal how MAGI3 exerts this function: through direct interaction with β-catenin’s C-terminal, MAGI3 promotes β-catenin phosphorylation at critical residues (phosphodegron) and subsequent ubiquitination, thereby targeting it for degradation (Fig. S6).

To elucidate how MAGI3 inhibits the Wnt/β-catenin signaling pathway, our findings revealed that MAGI3 promoted β-catenin phosphorylation and subsequent ubiquitin-mediated proteasomal degradation. Mechanistically, MAGI3 enhances the interaction between GSK3β and β-catenin by binding directly to the carboxyl terminus of β-catenin, facilitating GSK3β-mediated phosphorylation at the residues Ser33, Ser37, and Thr41 (Figs. 4, 5).

Through integrated structural and biochemical analyses, we demonstrate that MAGI3 binding triggers a conformational switch in β-catenin (Figs. 4, 5). Critically, this remodeling dissociates intramolecular constraints between the N-terminal tail and central Armadillo (ARM) repeat domain, thereby exposing cryptic phosphorylation motifs for GSK3β accessibility. Subsequent phosphorylation of these degrons initiates ubiquitin-mediated proteasomal degradation – a key regulatory event controlling metastasis-associated signaling cascades. Crucially, this MAGI3-dependent mechanism requires an intact C-terminal binding interface on β-catenin. Collectively, these results provide the first mechanistic evidence establishing MAGI3 as a molecular scaffold that controls β-catenin stability through targeted structural remodeling.

Previous study established that β-catenin lacking its C-terminal domain (ΔC-tail) undergoes accelerated turnover due to disrupted N-tail positioning [40]. While these studies demonstrated C-tail peptides competitively inhibit ARM domain binding to axin and thereby modulating scaffold complex accessibility, the conformational impact on N-terminal phosphodegron exposure remained unresolved. Our structural analyses now reveal MAGI3 binding to the β-catenin C-tail (residues 667–781) allosterically displaces the N-tail from the ARM domain (Fig. 4F–H), phenocopying ΔC-tail-induced structural destabilization. This spatial rearrangement exposes the Ser33/Ser37/Thr41 phosphodegron, enhancing GSK3β phosphorylation (Fig. 5F, G). This refined molecular perspective integrates earlier research findings and provides a deeper understanding of the regulatory mechanisms governing β-catenin stability. Building on prior work, our findings reveal novel mechanistic details underlying the regulation of β-catenin stability. Our previous work established MAGI3 as an E3 ubiquitin ligase that destabilizes c-Myc through dual-domain engagement: PDZ5 binds c-Myc while PDZ2 recruits SKP1 to assemble an SCF-like complex [28]. Strikingly, the current study reveals a mechanistically distinct paradigm for β-catenin regulation. Structural mapping demonstrates MAGI3’s PDZ4 domain (residues 721–815) as the primary β-catenin interface [31], a finding corroborated by AlphaFold-predicted hydrophobic packing between PDZ4’s β-sheet groove and β-catenin’s C-terminal PDZ-binding motif (Fig. 5A–E). Collectively, these structural insights, combined with our functional data, indicate that MAGI3 employs divergent strategies to control these two critical oncoproteins. It directly catalyzes c-Myc ubiquitination and degradation as an E3 ligase, while for β-catenin, it indirectly promotes proteasomal turnover. MAGI3 achieves the latter by facilitating β-catenin phosphorylation at Ser33, Ser37, and Thr41 through GSK3β.

Resistance to everolimus presents a major clinical challenge following immunotherapy and TKI treatment, with limited subsequent options available for patients with ccRCC [41, 42]. To elucidate the mechanisms underlying mTOR inhibitor resistance in ccRCC, we established complementary in vitro models and identified MAGI3 as a key determinant of everolimus sensitivity. Mechanistically, MAGI3 exerts this effect through suppression of the Wnt/β-catenin pathway. Consistent with this, MAGI3 knockdown significantly decreased cellular sensitivity to everolimus, as evidenced by a 4.8- to 6.9-fold increase in the IC50 value (from 1.82 μM to 8.66 μM and from 1.00 μM to 6.90 μM in two different cell lines; Fig. 7A). Furthermore, MAGI3 depletion led to constitutive activation of the Wnt/β-catenin pathway, establishing this pathway crosstalk as a key driver of resistance. Based on this mechanistic insight, we hypothesized that co-targeting both pathways would overcome resistance. Indeed, combined inhibition of mTOR and Wnt/β-catenin signaling using everolimus and XAV-939 demonstrated strong synergistic anti-tumor activity in vitro, effectively suppressing ccRCC cell proliferation and clonogenic survival (Fig. 7B–E).

Critically, this therapeutic strategy was validated in vivo using a xenograft model that recapitulates the MAGI3-low, therapy-resistant phenotype. Tumors with MAGI3 knockdown exhibited marked resistance to everolimus monotherapy, mirroring the clinical challenge. While XAV-939 monotherapy showed modest efficacy, the combination of everolimus and XAV-939 synergistically and potently inhibited tumor growth (Fig. 8F–H). Importantly, this enhanced anti-tumor effect was achieved without significant systemic toxicity, as indicated by stable body weights and the absence of overt adverse events (Fig. 8I). These in vivo findings not only reinforce the causal role of the MAGI3-β-catenin axis in driving everolimus resistance but also provide compelling preclinical evidence that dual-pathway blockade is a viable and well-tolerated strategy to overcome this resistance.

Translating these findings to clinical practice, we analyzed pre-treatment specimens from 53 ccRCC patients undergoing Everolimus therapy. Quantitative immunohistochemical analysis revealed significantly higher MAGI3 expression levels in responders compared to non-responders (*p* < 0.05), while β-catenin expression showed an inverse correlation (*p* < 0.05). Importantly, we observed a strong negative correlation between MAGI3 and β-catenin immunostaining scores (*r* = –0.63, *p* < 0.001; Fig. 8A–D). Clinical outcome analysis demonstrated striking prognostic significance: patients with low MAGI3 expression had significantly shorter overall survival than those with high expression (median OS 14 vs 22 months, HR = 2.65, 95% CI 1.248–5.609; log-rank *p* < 0.05; Fig. 8E). Taken together, these results support the conclusion that MAGI3-mediated inhibition of Wnt/β-catenin signaling plays a pivotal role in sustaining rapalog sensitivity in ccRCC.

The PI3K/AKT/mTOR and Wnt/β-catenin signaling pathways are known to engage in extensive crosstalk across various cancers, including colorectal, pancreatic, and breast cancers, as well as hepatocellular carcinoma and glioblastoma. These pathways share common effector molecules and are capable of mutual regulation, often leading to compensatory activation wherein the inhibition of one pathway results in the hyperactivation of the other. For instance, in colorectal cancer, blockade of the PI3K/AKT/mTOR axis can provoke aberrant Wnt/β-catenin signaling as a feedback mechanism to sustain cell survival [43]. The intricate reciprocity between Wnt/β-catenin and PI3K/AKT/mTOR pathways constitutes a core resistance axis in ccRCC. PI3K inhibition triggers compensatory Wnt hyperactivation—a pharmacodynamic escape mechanism documented in in vivo models [32, 44]. This feedback loop enables β-catenin to override mTOR blockade-induced apoptosis [43], explaining the ≤20% objective response rate to monotherapies in advanced ccRCC [45, 46]. Critically, our findings demonstrate that co-inhibition of AKT/mTOR and Wnt/β-catenin pathways overcomes these compensatory resistance mechanisms in preclinical models, revealing potent combined therapeutic potential for advanced ccRCC.

In conclusion, we establish MAGI3 as a clinically significant ccRCC tumor suppressor via its unique regulation of β-catenin stability. As a key modulator of Wnt/β-catenin signaling, MAGI3 provides the mechanistic basis for sustained rapalog sensitivity. Our work not only identifies key molecular drivers of resistance but directly informs novel dual-pathway therapeutic strategies.

## Supplementary information

supplement Figures

supplement Table 1–2

supplement methods Table 1–2

uncropped western blots

A Video Comparison of β-Catenin Conformation Before and After MAGI3 Interaction

## Data Availability

The previously published data are openly available in [TCGA, Kidney Renal Clear Cell Carcinoma, PanCancer Atlas] at [https://www.cbioportal.org/study/summary?id=kirc_tcga_pan_can_atlas_2018]; [GSE73731] at [https://www.ncbi.nlm.nih.gov/geo/query/acc.cgi?acc=GSE73731]. [GSE12606] at [https://www.ncbi.nlm.nih.gov/geo/query/acc.cgi?acc=GSE12606]. [GSE31232] at [https://www.ncbi.nlm.nih.gov/geo/query/acc.cgi?acc=GSE31232]. [GSE36895] at [https://www.ncbi.nlm.nih.gov/geo/query/acc.cgi?acc=GSE36895]. [GSE22541] at [https://www.ncbi.nlm.nih.gov/geo/query/acc.cgi?acc=GSE22541].
