# Supplementary material for: MAGI3 deficiency unleashes β-catenin conformational change to drive metastatic progression and mTOR inhibitor resistance in ccRCC
Source: Cell Death Dis. 2026 Mar 24;17(1):372. doi: 10.1038/s41419-026-08563-x (PMC13039909; doi:10.1038/s41419-026-08563-x)
Supplement: Supplementary file 1 — supplement Figures [file 41419_2026_8563_MOESM1_ESM.pdf]

Fig. S1

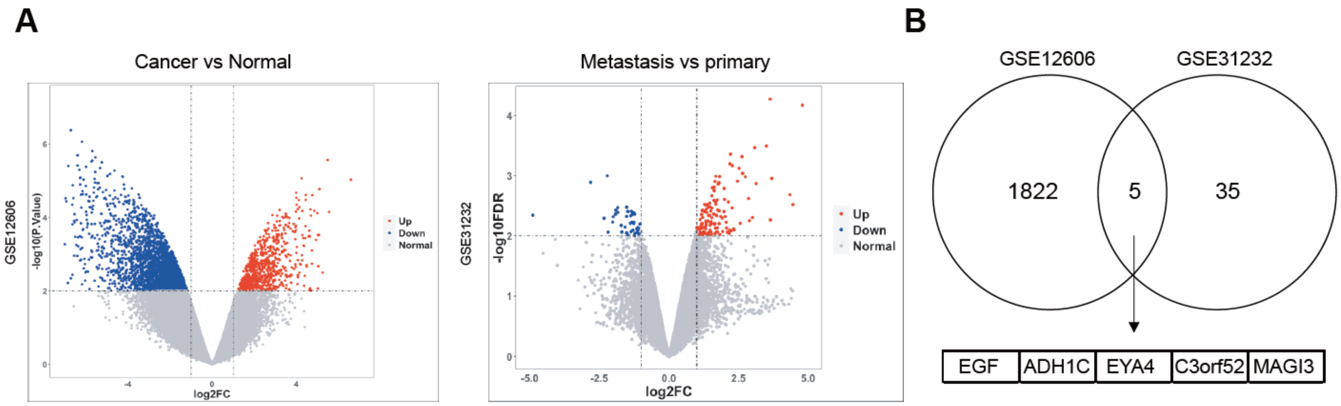

Fig. S1. Identification of Common Differentially Expressed Genes (DEGs) in ccRCC and mRCC Tissues.

(A)The volcano plot illustrates DEGs associated with ccRCC carcinogenesis and metastasis. DEGs from tumor vs. adjacent noncancerous tissues, and metastatic vs. nonmetastatic tumors in ccRCC patients from GSE12606 and GSE31232 datasets are depicted. The horizontal gray line represents a P value of 0.01, while the vertical gray lines indicate 1-fold changes in gene expression. Upregulated genes are denoted by red dots, and downregulated genes are represented by blue dots.

(B) The Venn diagram showcases the overlap of down-regulated DEGs in the GSE12606 and GSE31232 datasets. Among these, MAGI3, ADH1C, EGF, EYA4, and C3orf52 emerge as common DEGs.

Fig. S2

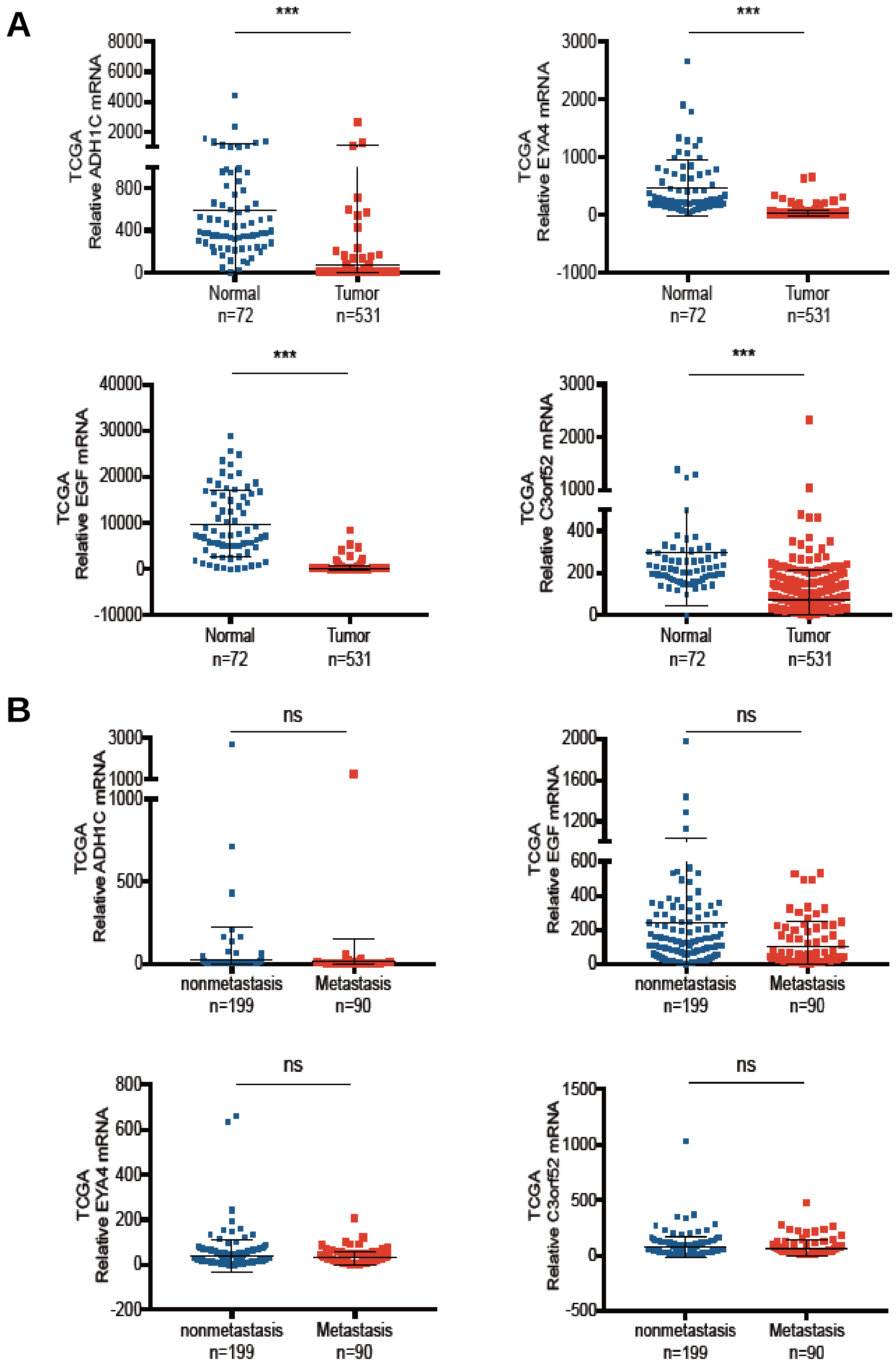

Fig. S2. The downregulation of ADH1C, EGF, EYA4, and C3orf52 expression in primary ccRCC, contrasting with their expression levels in metastatic tumors.

(A) Scatter plots depict the mRNA expression levels of ADH1C, EGF, EYA4, and C3orf52 in ccRCC and adjacent normal tissues from TCGA. *** $p < 0.001$, determined by independent sample t-test.

(B) Scatter plots showing the mRNA expression levels of ADH1C, EGF, EYA4, and C3orf52 in primary tumors compared to metastatic tumors. No significant difference is indicated by "ns". The values are presented as mean ± SD.

Fig. S3

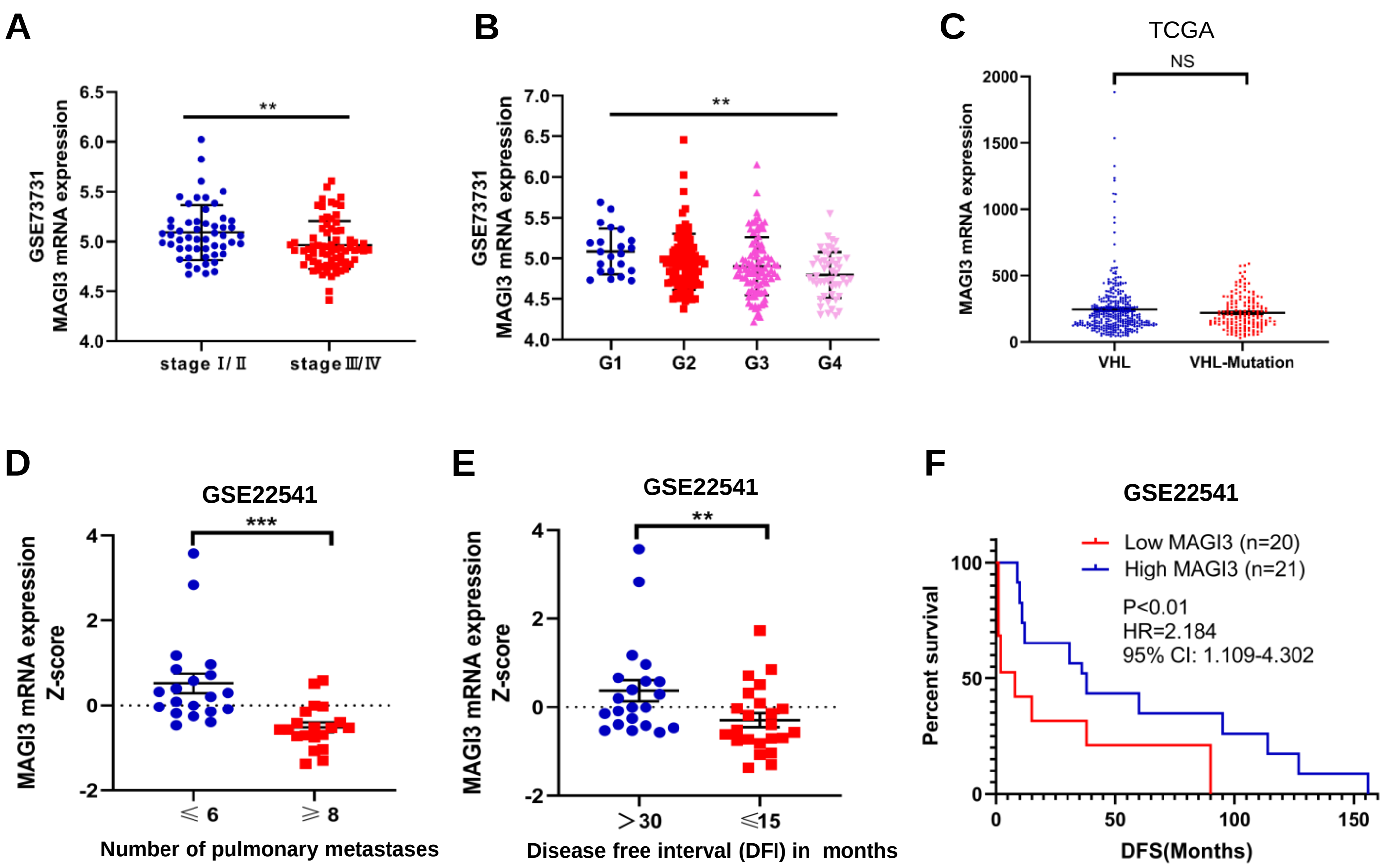

Fig. S3. Downregulation of MAGI3 expression in ccRCC patients with advanced stage, high grade, metastatic burden and disease progression.

(A) Scatter plots illustrate MAGI3 mRNA expression in ccRCC tissues with grades 1-4 from the GSE73731 dataset. ** $p < 0.01$, as determined by independent sample t-test.

(B) Scatter plots depict MAGI3 mRNA expression in ccRCC tissues with stage I/II and stage III/IV from the GSE73731 dataset. ** $p < 0.01$, as determined by independent sample t-test.

(C) Scatter plots depict MAGI3 mRNA expression in ccRCC tissues with VHL wildtype and VHL mutation from the TCGA dataset.

(D) MAGI3 expression levels in lung metastatic lesions (laser resection) from ccRCC patients with high (≥8 nodules) versus low (≤6 nodules) metastatic burden, from the GSE22541 cohort.

(E) Comparison of MAGI3 expression in lung metastases from patients with short (≤15 months) versus long (>30 months) disease-free intervals (DFI) from the GSE22541 cohort.

(F) Kaplan–Meier analysis of progression-free survival (PFS) stratified by high versus low MAGI3 expression in lung metastatic tissues from the GSE22541 cohort. Statistical comparisons were performed using the Mann–Whitney U test for D and E, and the log-rank test for F. *$P < 0.05$, **$P < 0.01$.

Fig. S4

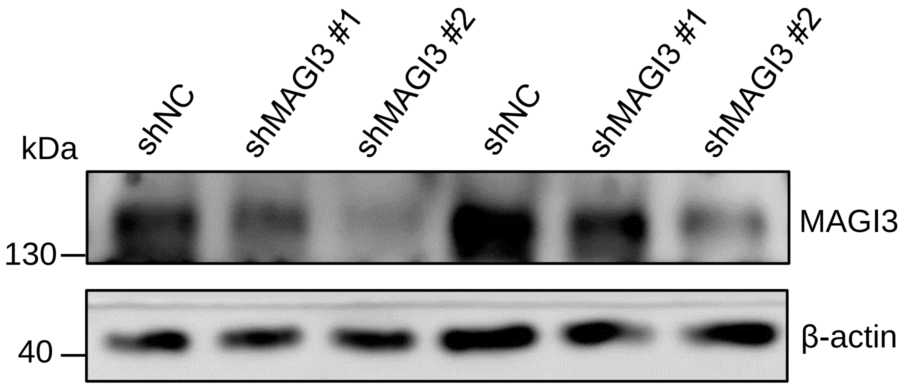

Fig. S4. Cell transfected with shNC, shMAGI3 #1 or shMAGI3 #, were subjected to western blotting analysis.

Fig. S5

A

TCGA

NAME	NES	NOM p-val	FDR q-val
KEGG_BASAL_TRANSCRIPTION_FACTORS	-1.990602	0.016935484	0.00720662
KEGG_WNT_SIGNALING_PATHWAY	-1.981953	0.0207635	0.0290351
KEGG_RNA_DEGRADATION	-1.654269	0.022044089	0.08401173
KEGG_OOCYTE_MEIOSIS	-1.639933	0.023076924	0.162115407
KEGG_TGF_BETA_SIGNALING_PATHWAY	-1.630642	0.011976048	0.25014965
KEGG_UBIQUITIN_MEDIATED_PROTEOLYSIS	-1.61554	0.017857144	0.45116407
KEGG_PROGESTERONE_MEDIATED_OOCYTE_MATURATION	-1.520554	0.05836576	0.6903304
KEGG_LONG_TERM_POTENTIATION	-1.509829	0.03314917	0.62778366
KEGG_ARRHYTHMOGENIC_RIGHT_VENTRICULAR_CARDIOMYOPATHY_ARVC	-1.480944	0.06654344	0.6475837
KEGG_HYPERTROPHIC_CARDIOMYOPATHY_HCM	-1.454469	0.051785715	0.6661896
KEGG_VASOPRESSIN_REGULATED_WATER_REABSORPTION	-1.434032	0.06786427	0.6722962
KEGG_ENDOMETRIAL_CANCER	-1.427063	0.072	0.63219166
KEGG_DILATED_CARDIOMYOPATHY	-1.380431	0.10344828	0.62425697
KEGG_CHRONIC_MYELOID_LEUKEMIA	-1.371212	0.09306931	0.6110265
KEGG_SPLICEOSOME	-1.337458	0.16	0.6653172

GSE36895

NAME	NES	NOM p-val	FDR q-val
KEGG_RIBOSOME	-2.02093	0	0.003770267
KEGG_CELL_CYCLE	-1.9813	0	0.024033722
KEGG_FOCAL_ADHESION	-1.97424	0	0.02773689
KEGG_WNT_SIGNALING_PATHWAY	-1.8719	0.008505468	0.01704145
KEGG_THYROID_CANCER	-1.7589	0.002832861	0.02676284
KEGG_OOCYTE_MEIOSIS	-1.74275	0	0.026694385
KEGG_UBIQUITIN_MEDIATED_PROTEOLYSIS	-1.6749	0	0.048279546
KEGG_SPLICEOSOME	-1.65899	0.001254705	0.050103307
KEGG_ARRHYTHMOGENIC_RIGHT_VENTRICULAR_CARDIOMYOPATHY_ARVC	-1.63903	0.001351351	0.05503813
KEGG_HYPERTROPHIC_CARDIOMYOPATHY_HCM	-1.59687	0.002649007	0.07325779
KEGG_LONG_TERM_POTENTIATION	-1.56695	0.00660502	0.08980175
KEGG_PROGESTERONE_MEDIATED_OOCYTE_MATURATION	-1.56184	0.009234829	0.085713334
KEGG_DILATED_CARDIOMYOPATHY	-1.53409	0.007822686	0.103122376
KEGG_CHRONIC_MYELOID_LEUKEMIA	-1.46379	0.025032938	0.16314308

B

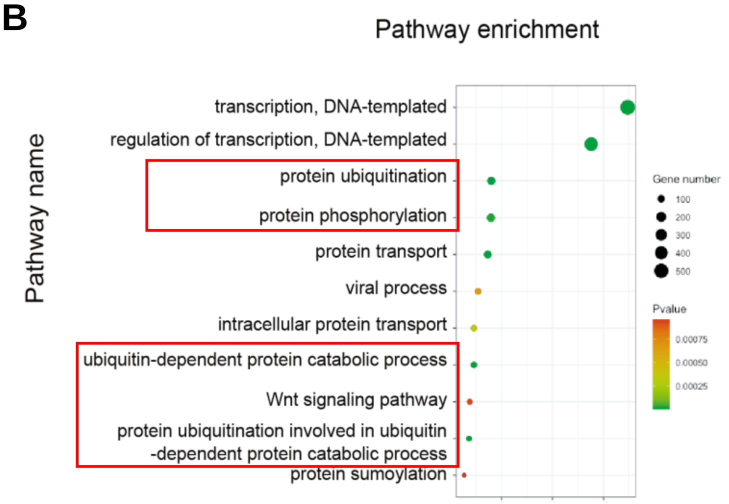

Fig. S5. MAGI3-related gene were predominantly enriched in pathways associated with ubiquitination dependent protein degradation and Wnt signaling pathway.

(A). Analysis results of gene sets enrichment in of high and low MAGI3 patients in TCGA and GSE36895 databases.

(B). The gene sets closely associated with MAGI3 expression in ccRCC were identified using KIRC data from TCGA via the UALCAN website, followed by pathway analysis for the enrichment of MAGI3-related genes conducted through DAVID.

Fig. S6

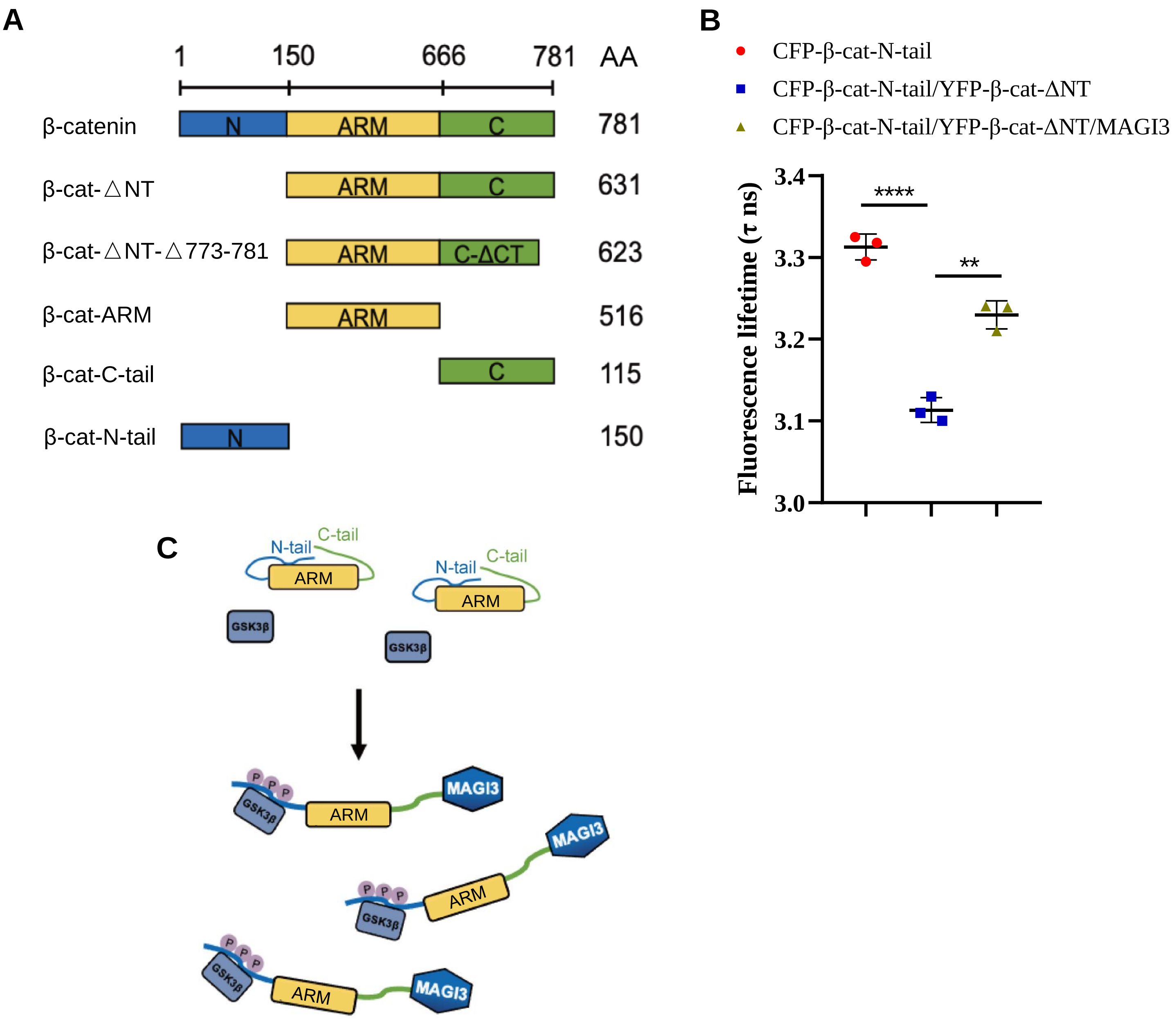

Fig. S6. MAGI3 regulates β-catenin phosphorylation and ubiquitination via C-tail interactions.

(A) Diagram illustrating the β-catenin GST fusion proteins, including full-length β-catenin (WT), N-tail, armadillo domain (ARM), C-tail, ΔNT (β-catenin with deleted N-tail), and ΔNT-ΔCT (β-catenin with deleted N-tail and C-terminus).

(B) Quantification of the average fluorescence lifetime of the donor (CFP-β-cat-N-tail) under the indicated co-expression conditions. Data are presented as mean ± SD. Statistical significance was determined by a one-way ANOVA with Tukey's multiple comparisons test. $^{*}p < 0.05$, $^{**}p < 0.01$, $^{***}p < 0.001$, $^{****}p < 0.0001$.

(C) Schematic representation illustrating the mechanism by which MAGI3 facilitates the release of β-catenin N-tail from its intramolecular interaction.

Fig. S7

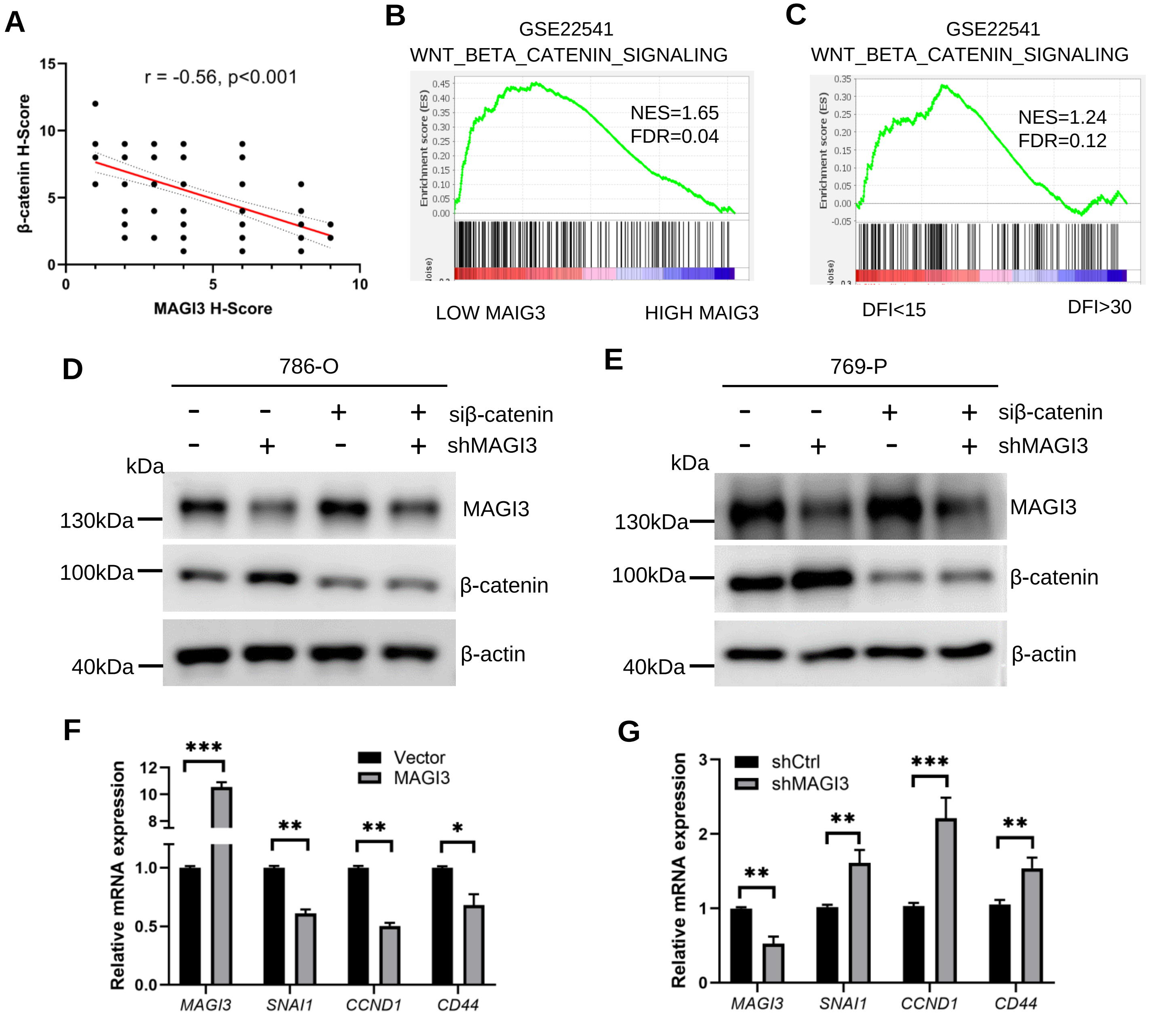

Fig. S7. MAGI3 plays a crucial role in suppressing metastasis in ccRCC cells by inhibiting β-catenin signaling

(A) Correlation analysis (Spearman) between MAGI3 H-score and β-catenin H-score in ccRCC specimens (n=119).

(B,C) Analysis of WNT_BETA_CATENIN_ gene sets enriched in ccRCC specimens with low MAGI3 expression and poor prognosis

(D, E). Knockdown of β-catenin rescues the increased β-catenin protein levels induced by MAGI3 knockdown. Cell transfected with shMAGI3 or siβ-catenin, were subjected to western blotting analysis.

(F, G). Quantitative real-time PCR (qRT-PCR) analysis of mRNA levels for established Wnt/β-catenin target genes (CCND1, SNAI1, CD44) in 786-O cells. Expression of target genes following stable overexpression of MAGI3 compared to empty vector control (F). Expression of target genes following stable knockdown of MAGI3 (shMAGI3) compared to scramble shRNA control (G). Data are presented as mean ± SD. Statistical significance was determined by Student's t-test (*p < 0.05, **p < 0.01, ***p < 0.001).

Fig. S8

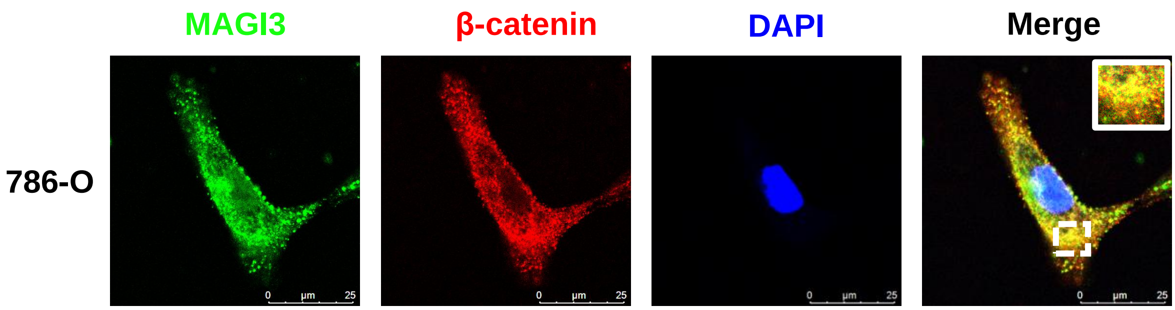

Fig.S8. MAGI3 specifically interacts with β-catenin at the cytoplasm.
Confocal microscopy images in 786-O cells, the MAGI3/β-catenin co-localization was visualized through merging of individual images. Colocalization of MAGI3 and β-catenin is shown in yellow at the cytoplasm. DAPI (blue) is used to stain the nucleus. Scale bars: 25 μm.
