# Supplementary material for: MAGI3 deficiency unleashes β-catenin conformational change to drive metastatic progression and mTOR inhibitor resistance in ccRCC
Source: Cell Death Dis. 2026 Mar 24;17(1):372. doi: 10.1038/s41419-026-08563-x (PMC13039909; doi:10.1038/s41419-026-08563-x)
Supplement: Supplementary file 2 — supplement Table 1–2 [file 41419_2026_8563_MOESM2_ESM.pdf]

Supplementary table 1. Univariate and multivariate analyses of MAGI3 mRNA level for patient OS

Variable	Univariate analysis		Multivariate analysis	
	P	HR（95% CI）	P	HR（95% CI）
**Overall survival (n = 39)**				
**Age (years)**				
＜60 (n = 15)				
⩾60 (n = 24)	0.532	1.374(0.507~3.726)		
**Grade**				
G1 or G2(n = 13)				
G3 or G4 (n = 26)	0.019	11.318(1.498~85.537)	0.630	1.878(0.145~24.358)
**MI**				
No(n = 16)				
Yes(n = 23)	0.031	3.955(1.135~13.776)	0.204	0.274(0.037~2.015)
**RVI**				
No(n = 23)				
Yes(n = 16)	0.062	2.521(0.956~6.648)		
**PI**				
No(n = 23)				
Yes(n = 16)	0.000	7.837(2.524~24.332)	0.668	1.460(0.258~8.260)
**SC**				
No(n = 31)				
Yes(n = 8)	0.007	4.612(1.525~13.944)	0.340	1.871(0.517~6.767)
**TN**				
No(n = 17)				
Yes(n = 22)	0.004	8.616(1.963~37.816)	0.049	7.906(1.011~61.843)
**T stage**				
T1 or T2 (n = 16)				
T3 or T4 (n = 23)	0.009	14.936(1.977~112.834)	0.104	15.517(0.569~423.413)
**N stage**				
N0 (n =31)				
N1 or N2(n =8)	0.004	4.342(1.610~11.713)	0.168	2.525(0.677~9.416)
**M stage**				
M0 (n =25)				
M1 (n =14)	0.031	2.908(1.102~7.678)	0.026	6.557(1.249~34.411)
**MAGI3**				
Low(n=18)				
High(n=21)	0.025	0.319(0.117~0.868)	0.006	0.080(0.013~0.478)

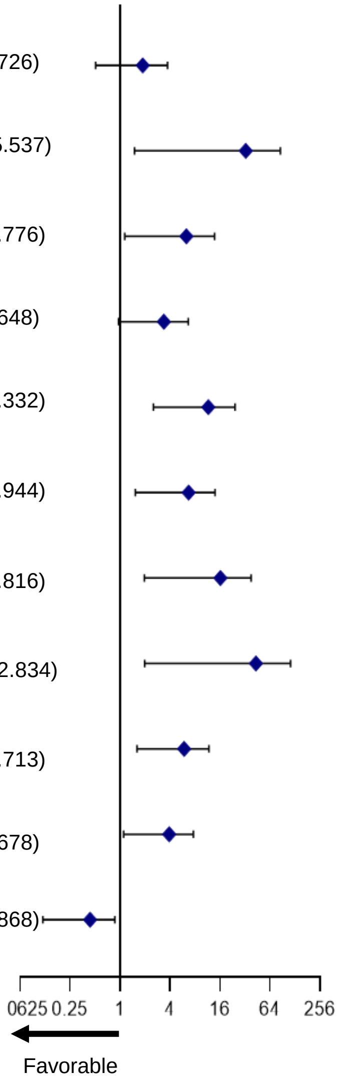

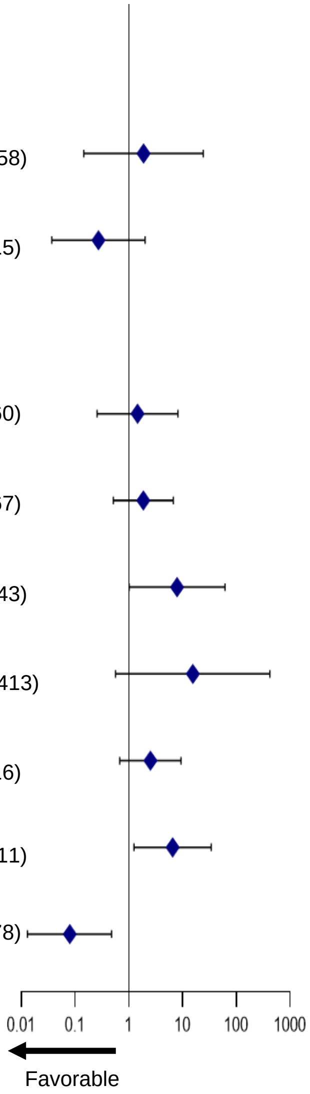

HR, hazard ratio; CI, confidence interval; MI, microvascular invasion; RVI, renal vein involvement; PI, peri-nephric fat involvement; SC, sarcomatoid component; TN, tumor necrosis

Supplementary table 2. Univariate and multivariate analyses of MAGI3 mRNA level for patient PFS

Variable	Univariate analysis P	Univariate analysis HR（95% CI）	Multivariate analysis P	Multivariate analysis HR（95% CI）
**Progression free survival (n = 39)**				
**Age (years)**				
＜60 (n = 15)				
⩾60 (n = 24)	0.325	1.581（0.635~3.932）		
**Grade**				
G1 or G2(n = 13)				
G3 or G4 (n = 26)	0.025	4.092（1.196~14.002）	0.725	0.745（0.144~3.850）
**MI**				
No(n = 16)				
Yes(n = 23)	0.007	5.362（1.576~18.244）	0.700	0.636（0.063~6.386）
**RVI**				
No(n = 23)				
Yes(n = 16)	0.021	2.858（1.175~6.950）	0.322	0.509（0.134~1.993）
**PI**				
No(n = 23)				
Yes(n = 16)	0.000	6.185（2.362~16.201）	0.393	1.849（0.451~7.576）
**SC**				
No(n = 31)				
Yes(n = 8)	0.001	6.211（2.112~18.266）	0.090	3.062（0.840~11.157）
**TN**				
No(n = 17)				
Yes(n = 22)	0.015	3.539（1.281~9.781）	0.184	2.308（0.673~7.918）
**T stage**				
T1 or T2 (n = 16)				
T3 or T4 (n = 23)	0.003	9.429（2.191~40.585）	0.199	6.484（0.373~112.707）
**N stage**				
N0 (n =31)				
N1 or N2(n =8)	0.007	3.671（1.417~9.512）	0.289	1.984（0.559~7.044）
**M stage**				
M0 (n =25)				
M1 (n =14)	0.015	3.138（1.251~7.872）	0.126	2.914（0.740~11.475）
**MAGI3**				
Low(n=18)				
High(n=21)	0.045	0.408（0.170~0.980）	0.025	0.229（0.063~0.833）

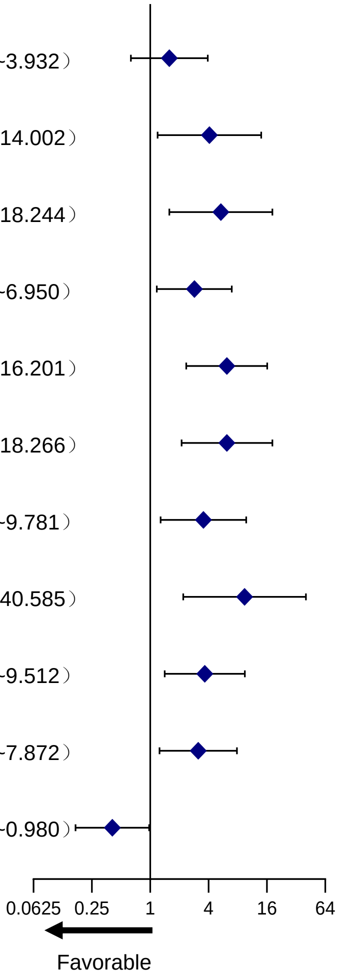

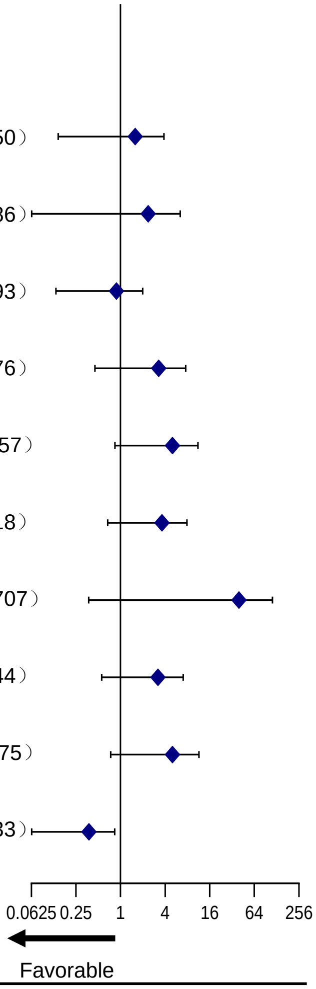

HR, hazard ratio; CI, confidence interval; MI, microvascular invasion; RVI, renal vein involvement; PI, peri-nephric fat involvement; SC, sarcomatoid component; TN, tumor necrosis
