# Supplementary material for: MAGI3 deficiency unleashes β-catenin conformational change to drive metastatic progression and mTOR inhibitor resistance in ccRCC
Source: Cell Death Dis. 2026 Mar 24;17(1):372. doi: 10.1038/s41419-026-08563-x (PMC13039909; doi:10.1038/s41419-026-08563-x)
Supplement: Supplementary file 3 — supplement methods Table 1–2 [file 41419_2026_8563_MOESM3_ESM.pdf]

Supplementary methods Table 1

Construct	Amino acids	Forward primer	Reverse primer
GST-β-cat-ΔNT	151-781	GGCCCCTGGGATCCCGTGCAATCCCTGA	GATGCGGCCGCTCGAGTTACAGGTCAG
GST-β-cat-ΔNT-Δ773-781	151-773	GGCCCCTGGGATCCCGTGCAATCCCTGA	GATGCGGCCGCTCGAGTTACTGATTGCTG
GST-β-cat-ARM	151-666	GGCCCCTGGGATCCCGTGCAATCCCTGA	GATGCGGCCGCTCGAGTTACTTGTCCTCAGAC
GST-β-cat-C-tail	667-781	GGGGCCCCTGGGATCCCCACAAGATTACAA	GATGCGGCCGCTCGAGTTACAGGTCAG
Flag-β-cat-N-tail	1-150	TTGCGGCCGCGAATTCTATGGCTACTCAAGC	ATGCCACCCGGGATCCTTATGTGGCAAGTTCTG

Supplementary methods Table 2

MAGI3	forward	5'-CCTCGGTCACACTCATGCAGATG-3 '
	reverse	5'-GGTAGCAGCAACAATGTCCACAAC-3'
CCND1	forward	5'- GACCTTCGTTGCCCTCTGTG -3'
	reverse	5'- CGTGTGAGGCGGTAGTAGGA -3'
SNAI1	forward	5'- CTCGCTGCCAATGCTCATCTG-3'
	reverse	5'- AGCCTTTCCCACTGTCCTCATC -3'
CD44	forward	5'- CCTACCCACCCCAATTTCAT-3'
	reverse	5'- GCAGTTGTTCTTTGCTCAGG -3'
