# Supplementary material for: MAGI3 deficiency unleashes β-catenin conformational change to drive metastatic progression and mTOR inhibitor resistance in ccRCC
Source: Cell Death Dis. 2026 Mar 24;17(1):372. doi: 10.1038/s41419-026-08563-x (PMC13039909; doi:10.1038/s41419-026-08563-x)

Figure 1E

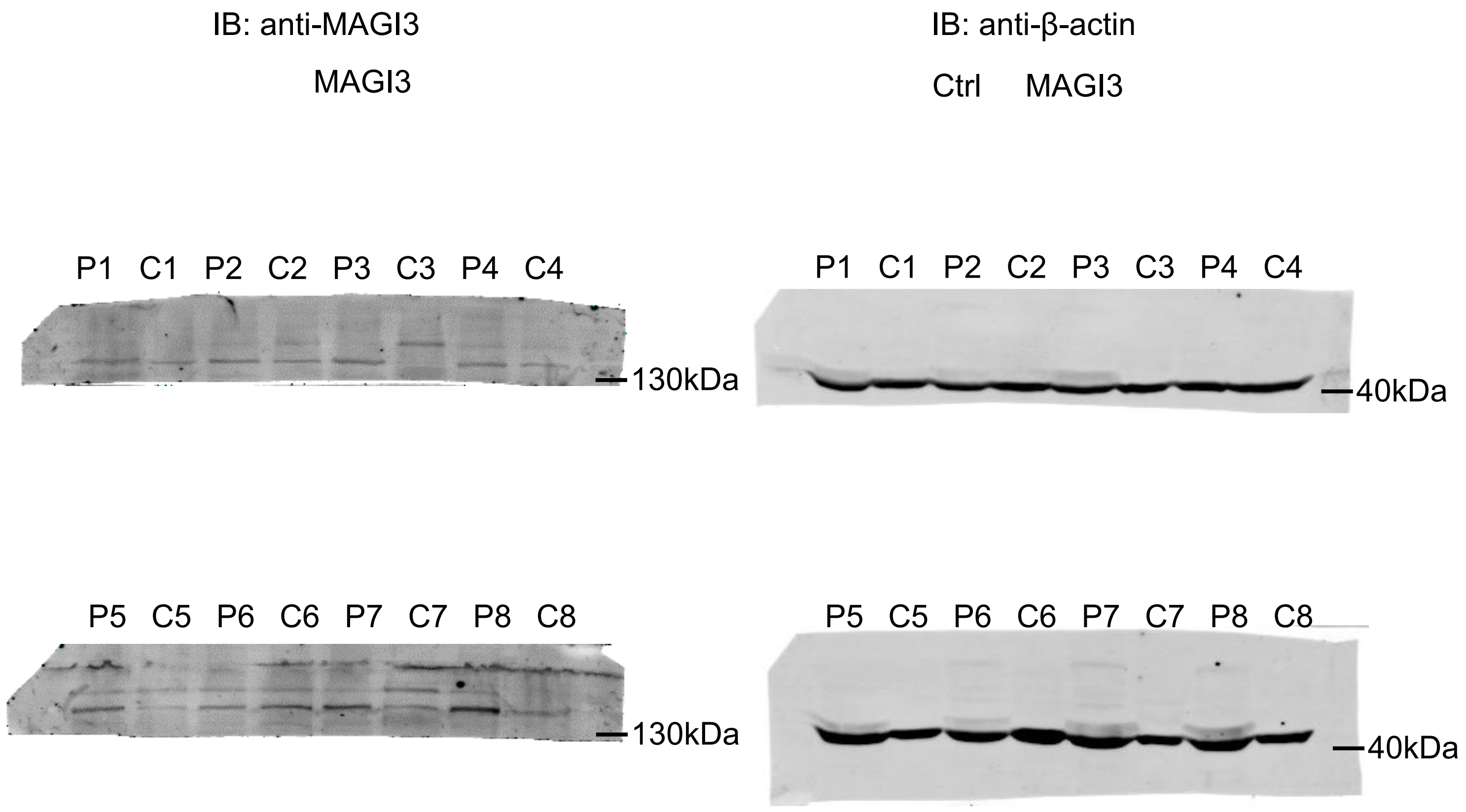

IB: anti-MAGI3
MAGI3
IB: anti-β-actin
Ctrl
MAGI3
P1 C1 P2 C2 P3 C3 P4 C4
130kDa
P1 C1 P2 C2 P3 C3 P4 C4
40kDa
P5 C5 P6 C6 P7 C7 P8 C8
130kDa
P5 C5 P6 C6 P7 C7 P8 C8
40kDa

Figure 2B

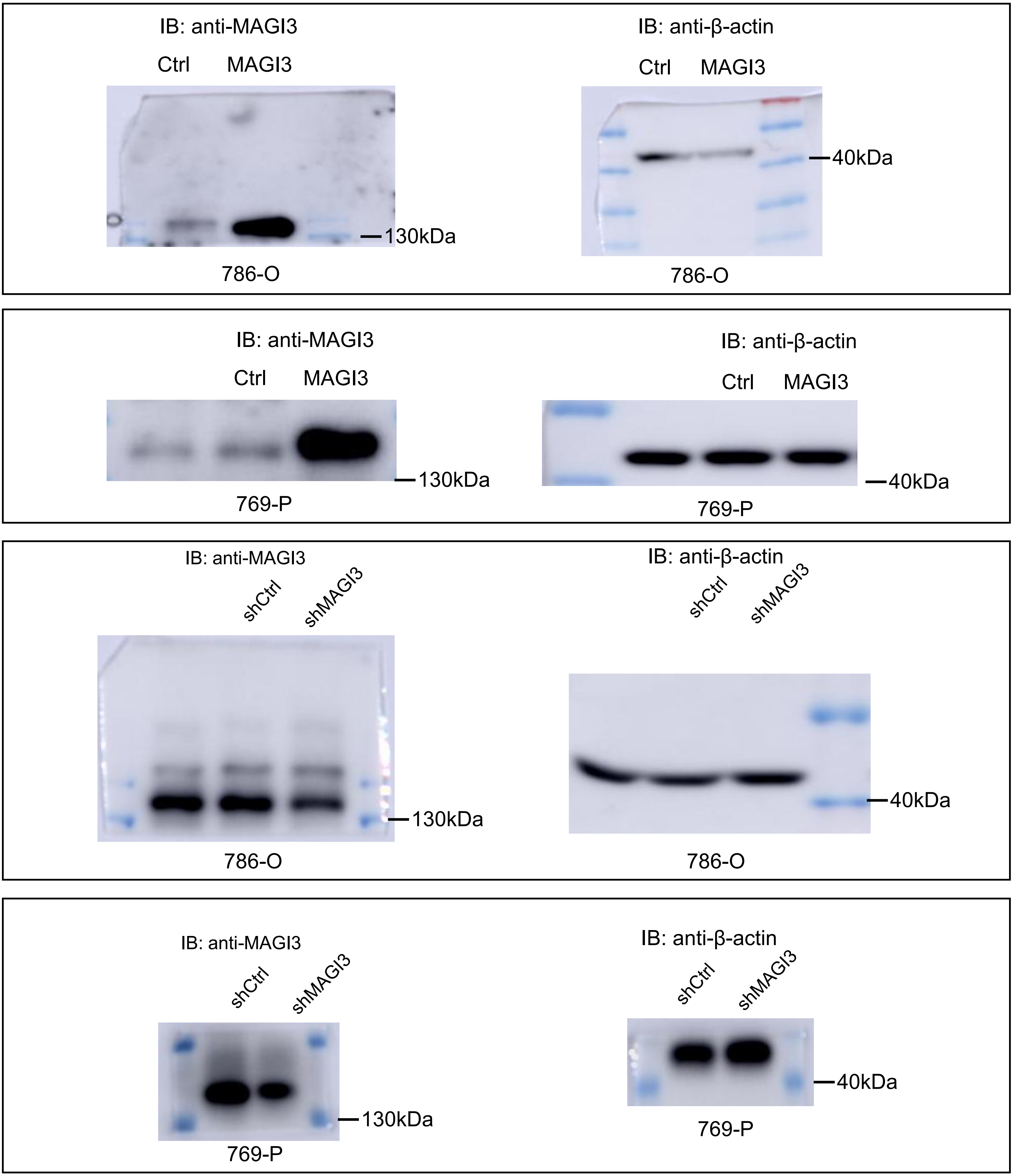
IB: anti-MAGI3
Ctrl
MAGI3
130kDa
786-O
IB: anti-β-actin
Ctrl
MAGI3
40kDa
786-O
IB: anti-MAGI3
Ctrl
MAGI3
130kDa
769-P
IB: anti-β-actin
Ctrl
MAGI3
40kDa
769-P
IB: anti-MAGI3
shCtrl
shMAGI3
130kDa
786-O
IB: anti-β-actin
shCtrl
shMAGI3
40kDa
786-O
IB: anti-MAGI3
shCtrl
shMAGI3
130kDa
769-P
IB: anti-β-actin
shCtrl
shMAGI3
40kDa
769-P

Figure 3D, E

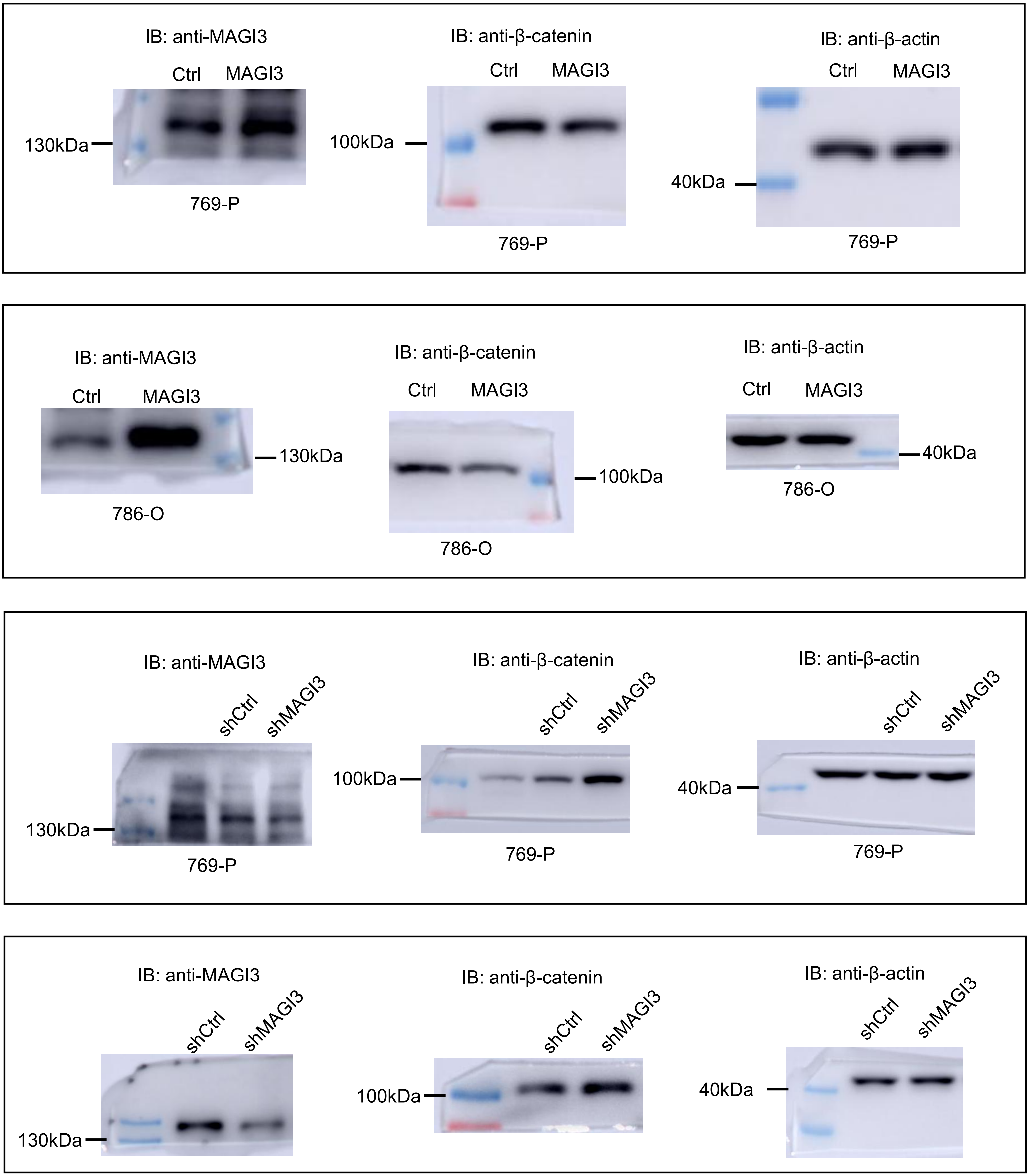
IB: anti-MAGI3
Ctrl
MAGI3
130kDa
769-P
IB: anti-β-catenin
Ctrl
MAGI3
100kDa
769-P
IB: anti-β-actin
Ctrl
MAGI3
40kDa
769-P
IB: anti-MAGI3
Ctrl
MAGI3
130kDa
786-O
IB: anti-β-catenin
Ctrl
MAGI3
100kDa
786-O
IB: anti-β-actin
Ctrl
MAGI3
40kDa
786-O
IB: anti-MAGI3
shCtrl
shMAGI3
130kDa
769-P
IB: anti-β-catenin
shCtrl
shMAGI3
100kDa
769-P
IB: anti-β-actin
shCtrl
shMAGI3
40kDa
769-P
IB: anti-MAGI3
shCtrl
shMAGI3
130kDa
IB: anti-β-catenin
shCtrl
shMAGI3
100kDa
IB: anti-β-actin
shCtrl
shMAGI3
40kDa

Figure 3F

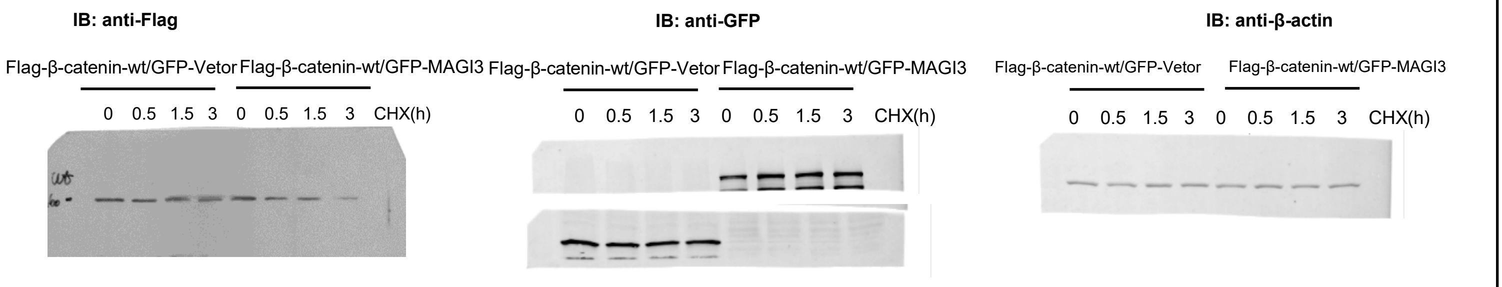

Figure 3G

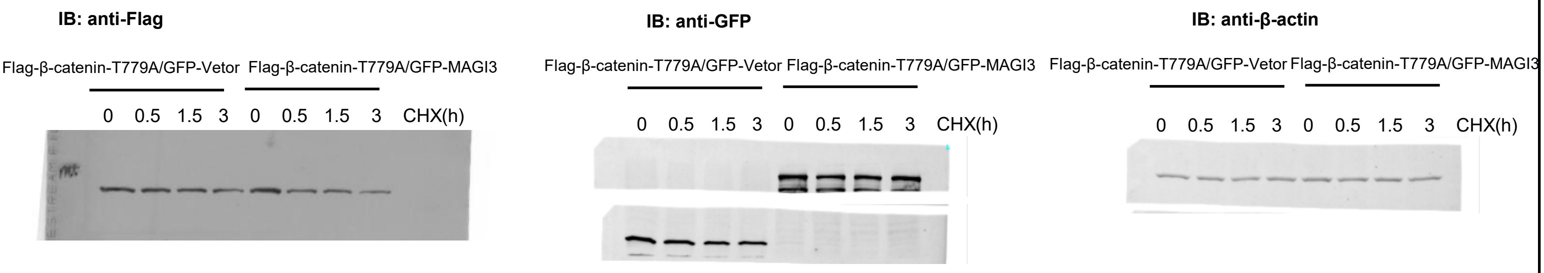

Figure 3H

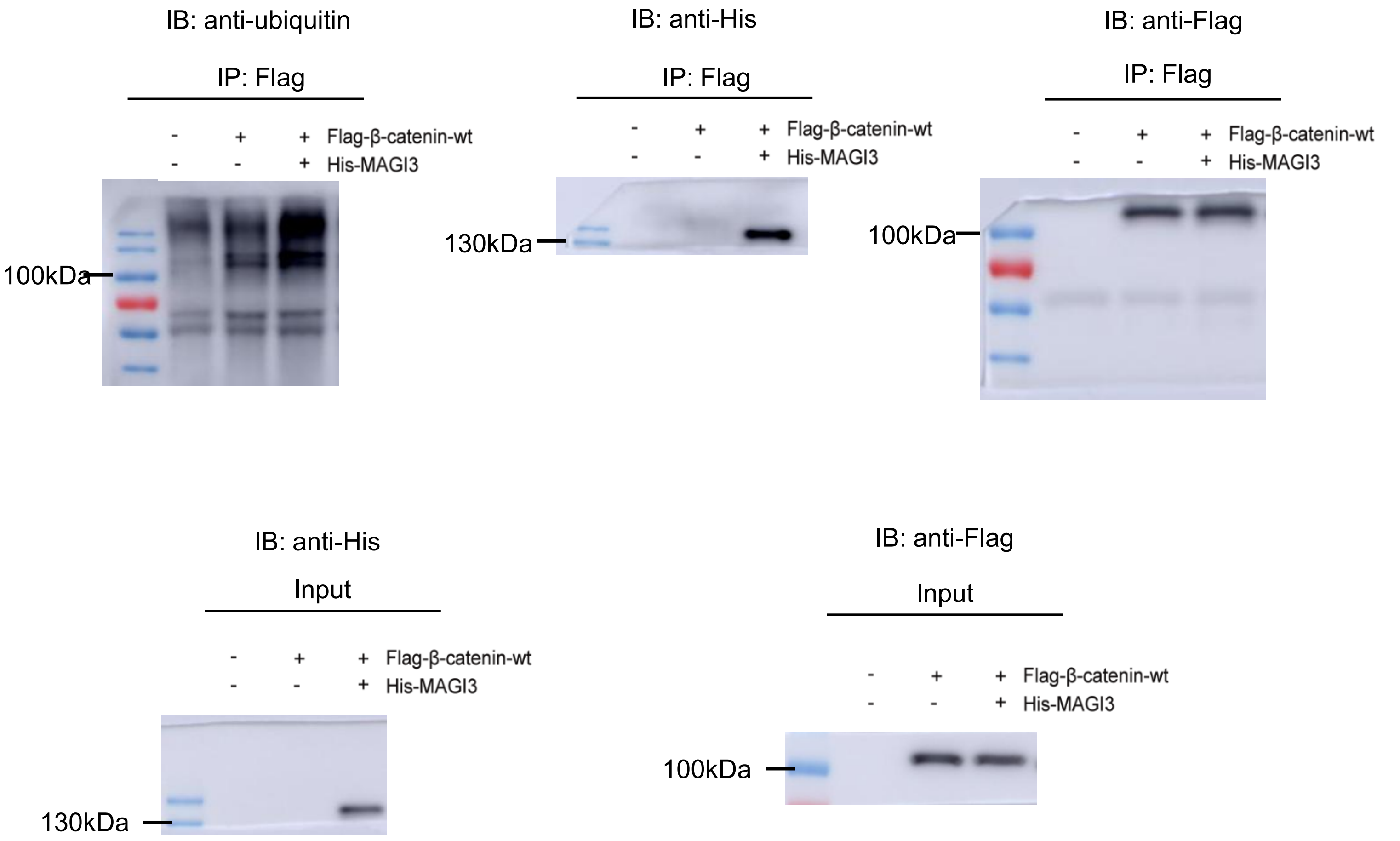
IB: anti-ubiquitin
IP: Flag
- + + Flag-β-catenin-wt
- - + His-MAGI3
100kDa
IB: anti-His
IP: Flag
- + + Flag-β-catenin-wt
- - + His-MAGI3
130kDa
IB: anti-Flag
IP: Flag
- + + Flag-β-catenin-wt
- - + His-MAGI3
100kDa
IB: anti-His
Input
- + + Flag-β-catenin-wt
- - + His-MAGI3
130kDa
IB: anti-Flag
Input
- + + Flag-β-catenin-wt
- - + His-MAGI3
100kDa

Figure 3I

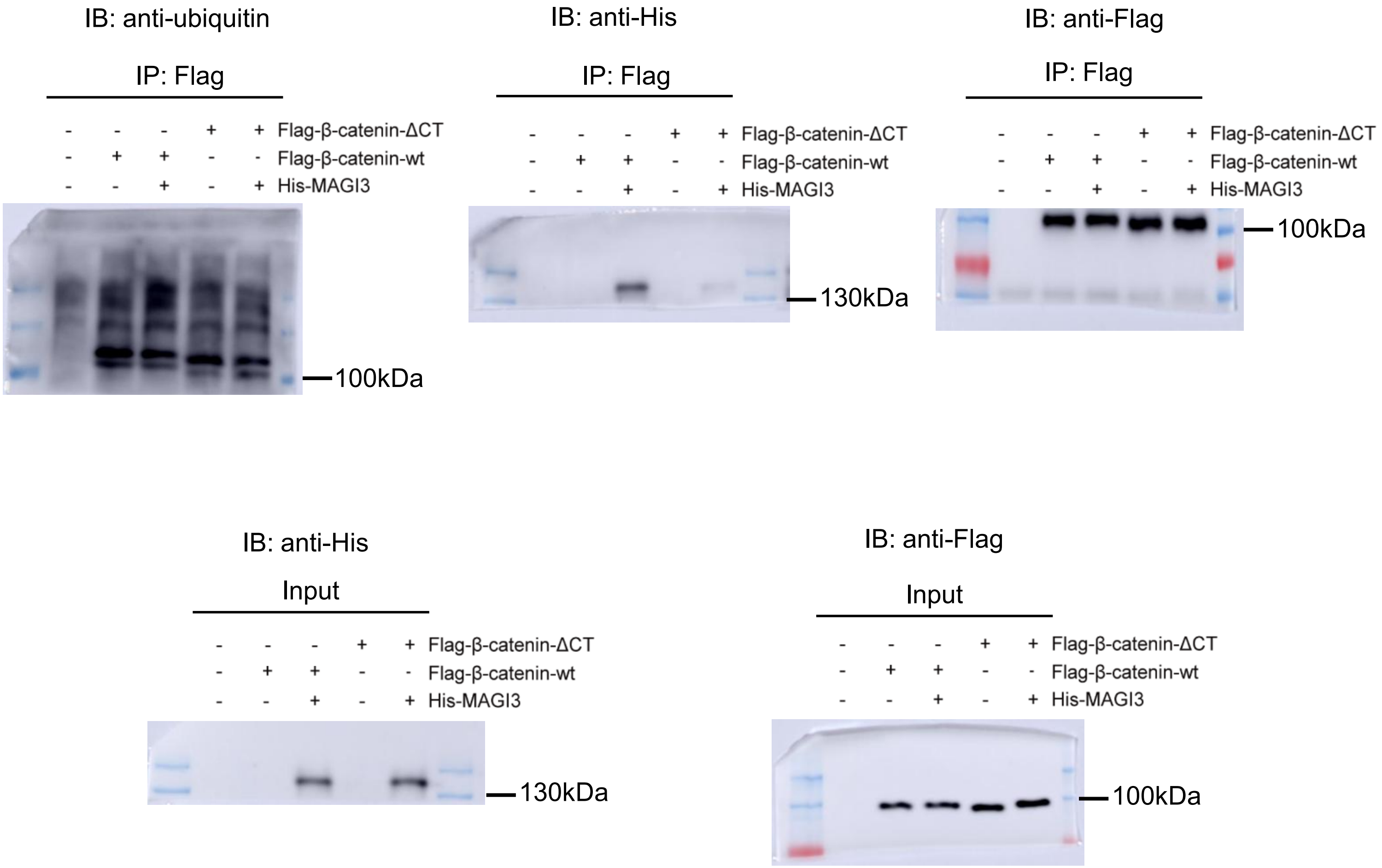

IB: anti-ubiquitin
IP: Flag
- - - + + Flag-β-catenin-ΔCT
- + + - - Flag-β-catenin-wt
- - + - + His-MAGI3
100kDa
IB: anti-His
IP: Flag
- - - + + Flag-β-catenin-ΔCT
- + + - - Flag-β-catenin-wt
- - + - + His-MAGI3
130kDa
IB: anti-Flag
IP: Flag
- - - + + Flag-β-catenin-ΔCT
- + + - - Flag-β-catenin-wt
- - + - + His-MAGI3
100kDa
IB: anti-His
Input
- - - + + Flag-β-catenin-ΔCT
- + + - - Flag-β-catenin-wt
- - + - + His-MAGI3
130kDa
IB: anti-Flag
Input
- - - + + Flag-β-catenin-ΔCT
- + + - - Flag-β-catenin-wt
- - + - + His-MAGI3
100kDa

Figure 4A, B

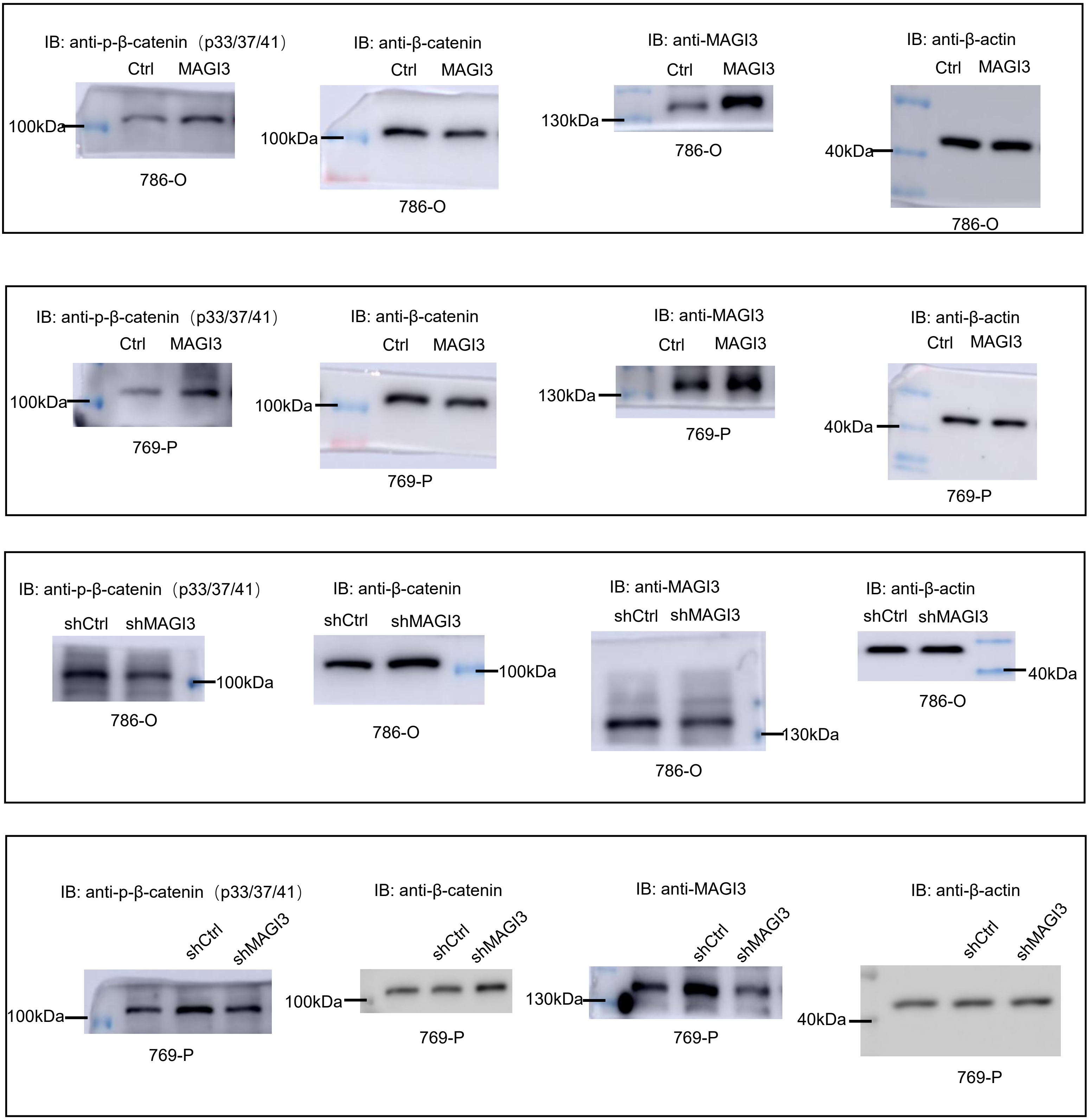
IB: anti-p-β-catenin（p33/37/41）
Ctrl MAGI3
100kDa
786-O
IB: anti-β-catenin
Ctrl MAGI3
100kDa
786-O
IB: anti-MAGI3
Ctrl MAGI3
130kDa
786-O
IB: anti-β-actin
Ctrl MAGI3
40kDa
786-O
IB: anti-p-β-catenin（p33/37/41）
Ctrl MAGI3
100kDa
769-P
IB: anti-β-catenin
Ctrl MAGI3
100kDa
769-P
IB: anti-MAGI3
Ctrl MAGI3
130kDa
769-P
IB: anti-β-actin
Ctrl MAGI3
40kDa
769-P
IB: anti-p-β-catenin（p33/37/41）
shCtrl shMAGI3
100kDa
786-O
IB: anti-β-catenin
shCtrl shMAGI3
100kDa
786-O
IB: anti-MAGI3
shCtrl shMAGI3
130kDa
786-O
IB: anti-β-actin
shCtrl shMAGI3
40kDa
786-O
IB: anti-p-β-catenin（p33/37/41）
shCtrl shMAGI3
100kDa
769-P
IB: anti-β-catenin
shCtrl shMAGI3
100kDa
769-P
IB: anti-MAGI3
shCtrl shMAGI3
130kDa
769-P
IB: anti-β-actin
shCtrl shMAGI3
40kDa
769-P

Figure 4C

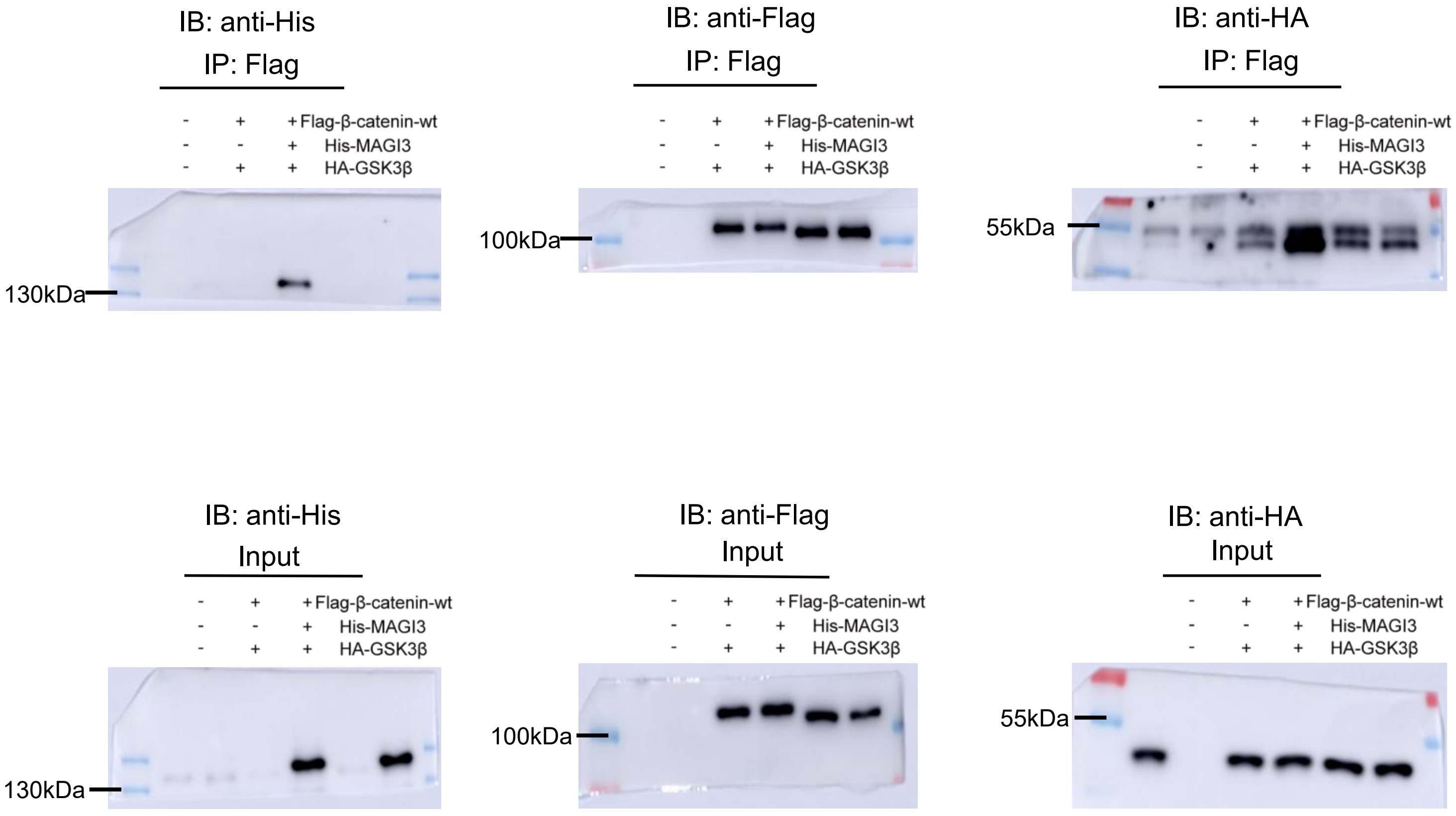
IB: anti-His
IP: Flag
- + + Flag-β-catenin-wt
- - + His-MAGI3
- + + HA-GSK3β
130kDa
IB: anti-Flag
IP: Flag
- + + Flag-β-catenin-wt
- - + His-MAGI3
- + + HA-GSK3β
100kDa
IB: anti-HA
IP: Flag
- + + Flag-β-catenin-wt
- - + His-MAGI3
- + + HA-GSK3β
55kDa
IB: anti-His
Input
- + + Flag-β-catenin-wt
- - + His-MAGI3
- + + HA-GSK3β
130kDa
IB: anti-Flag
Input
- + + Flag-β-catenin-wt
- - + His-MAGI3
- + + HA-GSK3β
100kDa
IB: anti-HA
Input
- + + Flag-β-catenin-wt
- - + His-MAGI3
- + + HA-GSK3β
55kDa

Figure 4D

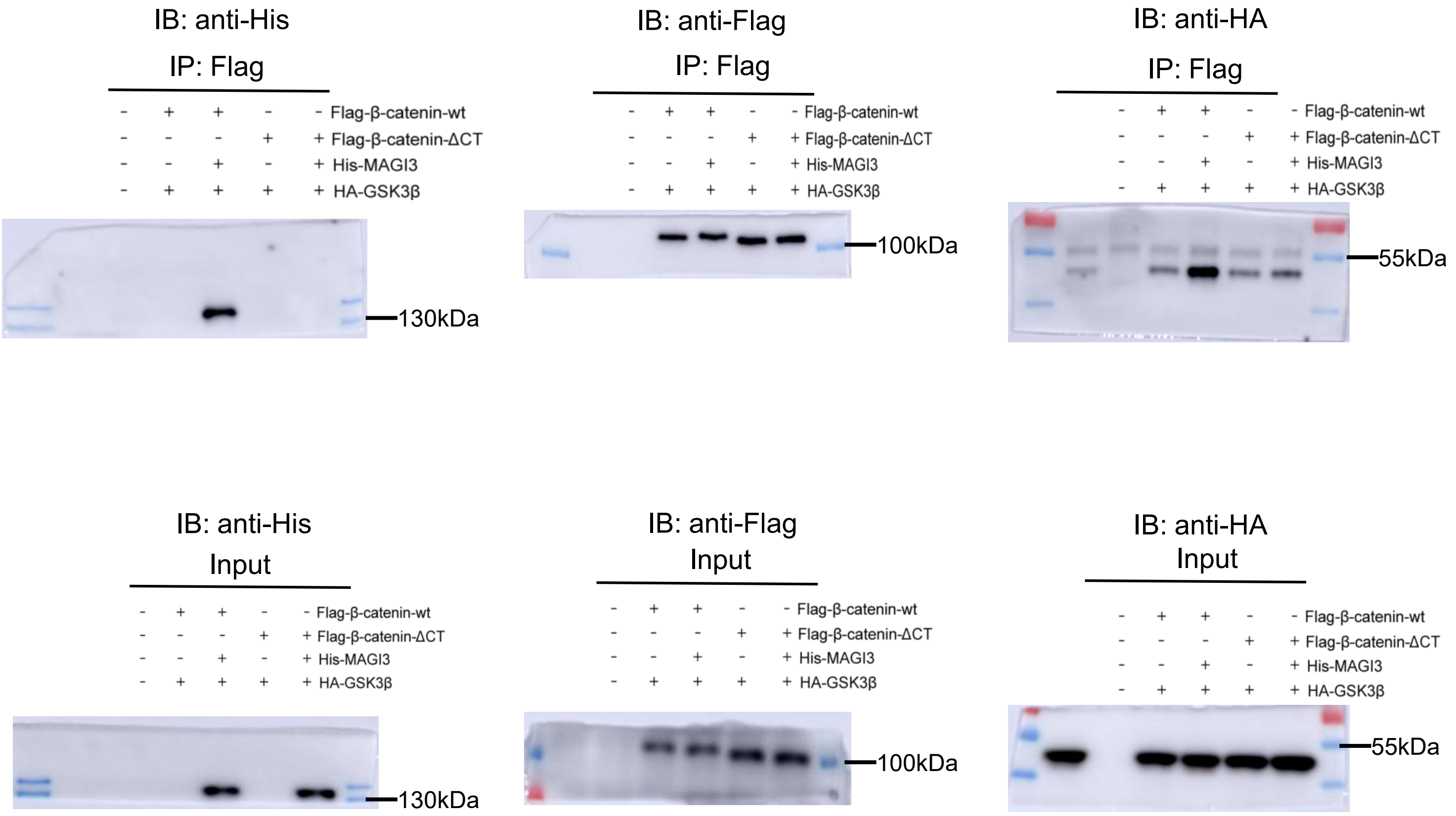
IB: anti-His
IP: Flag
- + + - - Flag-β-catenin-wt
- - - + + Flag-β-catenin-ΔCT
- - + - + His-MAGI3
- + + + + HA-GSK3β
130kDa
IB: anti-Flag
IP: Flag
- + + - - Flag-β-catenin-wt
- - - + + Flag-β-catenin-ΔCT
- - + - + His-MAGI3
- + + + + HA-GSK3β
100kDa
IB: anti-HA
IP: Flag
- + + - - Flag-β-catenin-wt
- - - + + Flag-β-catenin-ΔCT
- - + - + His-MAGI3
- + + + + HA-GSK3β
55kDa
IB: anti-His
Input
- + + - - Flag-β-catenin-wt
- - - + + Flag-β-catenin-ΔCT
- - + - + His-MAGI3
- + + + + HA-GSK3β
130kDa
IB: anti-Flag
Input
- + + - - Flag-β-catenin-wt
- - - + + Flag-β-catenin-ΔCT
- - + - + His-MAGI3
- + + + + HA-GSK3β
100kDa
IB: anti-HA
Input
- + + - - Flag-β-catenin-wt
- - - + + Flag-β-catenin-ΔCT
- - + - + His-MAGI3
- + + + + HA-GSK3β
55kDa

Figure 4E

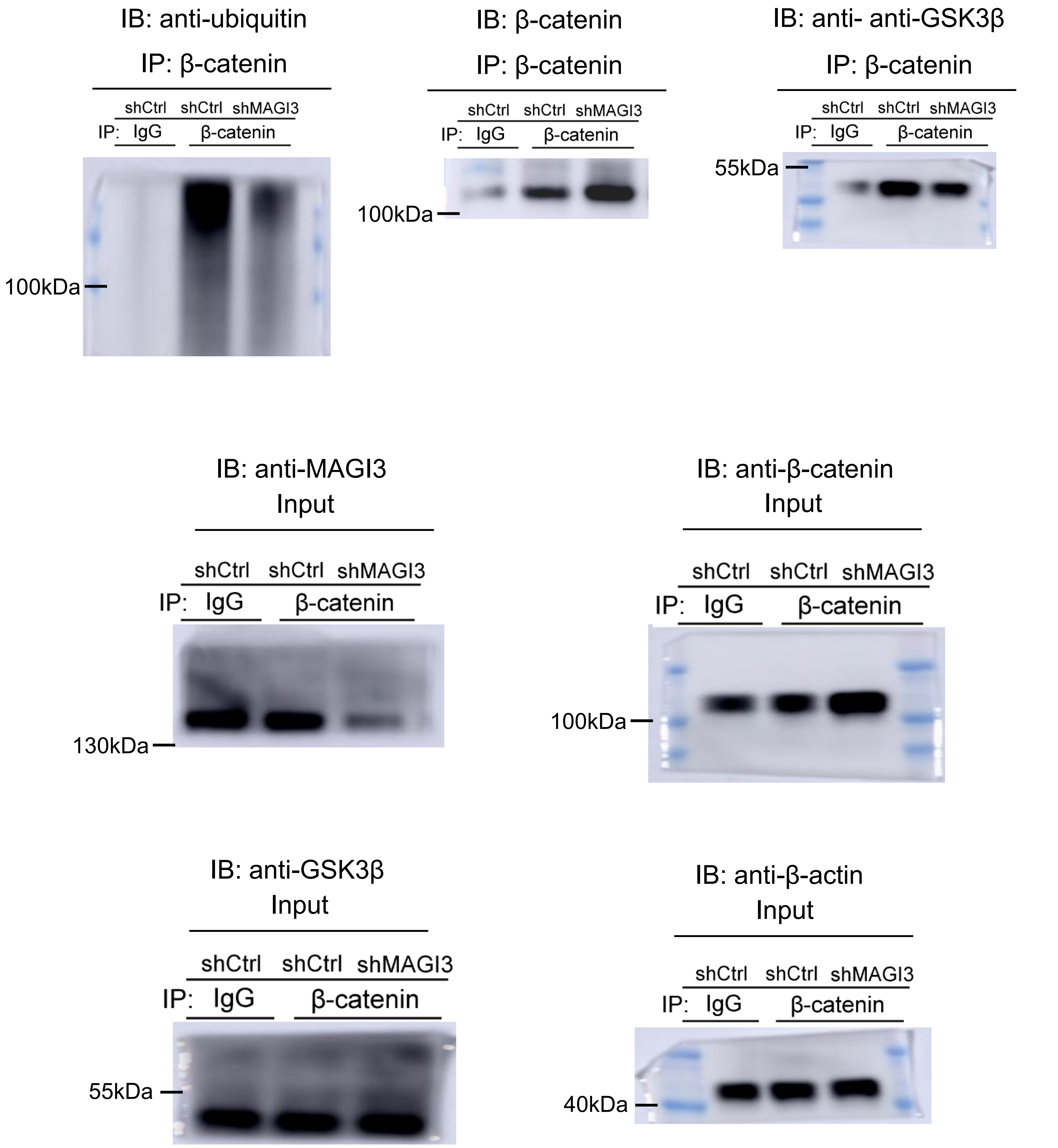
IB: anti-ubiquitin
IP: β-catenin
shCtrl shCtrl shMAGI3
IP: IgG β-catenin
100kDa
IB: β-catenin
IP: β-catenin
shCtrl shCtrl shMAGI3
IP: IgG β-catenin
100kDa
IB: anti- anti-GSK3β
IP: β-catenin
shCtrl shCtrl shMAGI3
IP: IgG β-catenin
55kDa
IB: anti-MAGI3
Input
shCtrl shCtrl shMAGI3
IP: IgG β-catenin
130kDa
IB: anti-β-catenin
Input
shCtrl shCtrl shMAGI3
IP: IgG β-catenin
100kDa
IB: anti-GSK3β
Input
shCtrl shCtrl shMAGI3
IP: IgG β-catenin
55kDa
IB: anti-β-actin
Input
shCtrl shCtrl shMAGI3
IP: IgG β-catenin
40kDa

Figure 4F

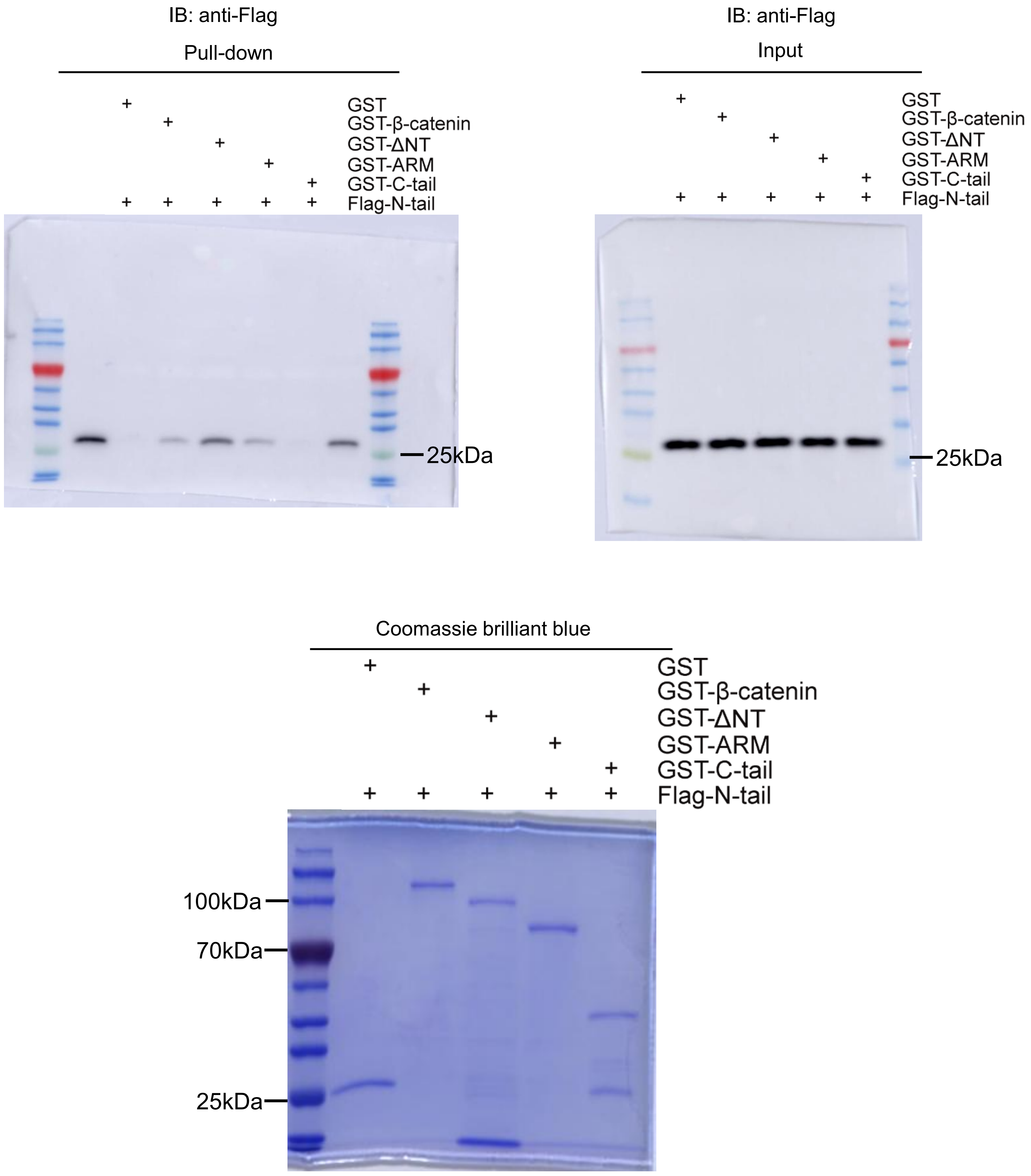
IB: anti-Flag
Pull-down
GST
GST-β-catenin
GST-ΔNT
GST-ARM
GST-C-tail
Flag-N-tail
25kDa
IB: anti-Flag
Input
GST
GST-β-catenin
GST-ΔNT
GST-ARM
GST-C-tail
Flag-N-tail
25kDa
Coomassie brilliant blue
GST
GST-β-catenin
GST-ΔNT
GST-ARM
GST-C-tail
Flag-N-tail
100kDa
70kDa
25kDa

Figure 4G

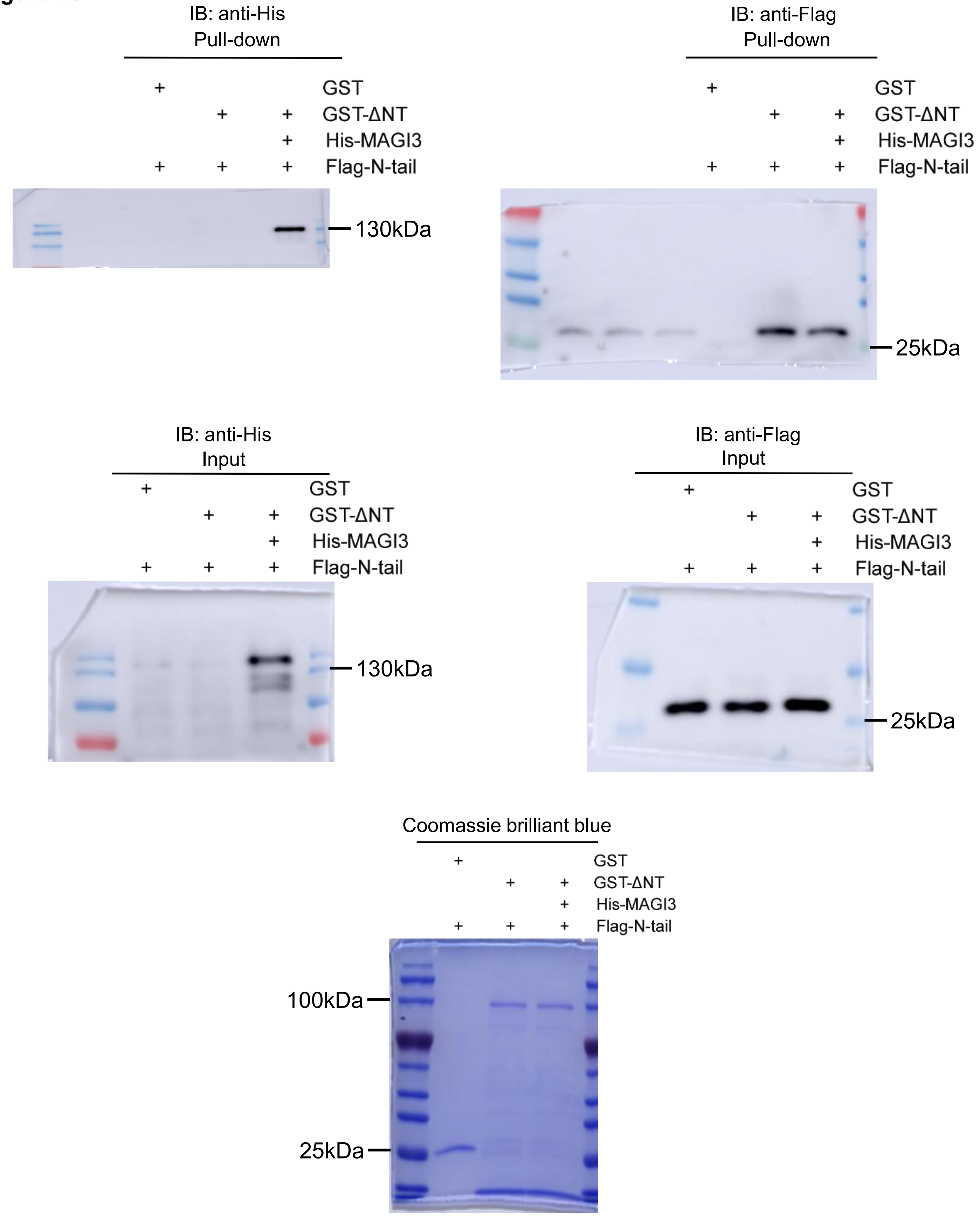

IB: anti-His
Pull-down
GST
GST-ΔNT
His-MAGI3
Flag-N-tail
130kDa
IB: anti-Flag
Pull-down
GST
GST-ΔNT
His-MAGI3
Flag-N-tail
25kDa
IB: anti-His
Input
GST
GST-ΔNT
His-MAGI3
Flag-N-tail
130kDa
IB: anti-Flag
Input
GST
GST-ΔNT
His-MAGI3
Flag-N-tail
25kDa
Coomassie brilliant blue
GST
GST-ΔNT
His-MAGI3
Flag-N-tail
100kDa
25kDa

Figure 4H

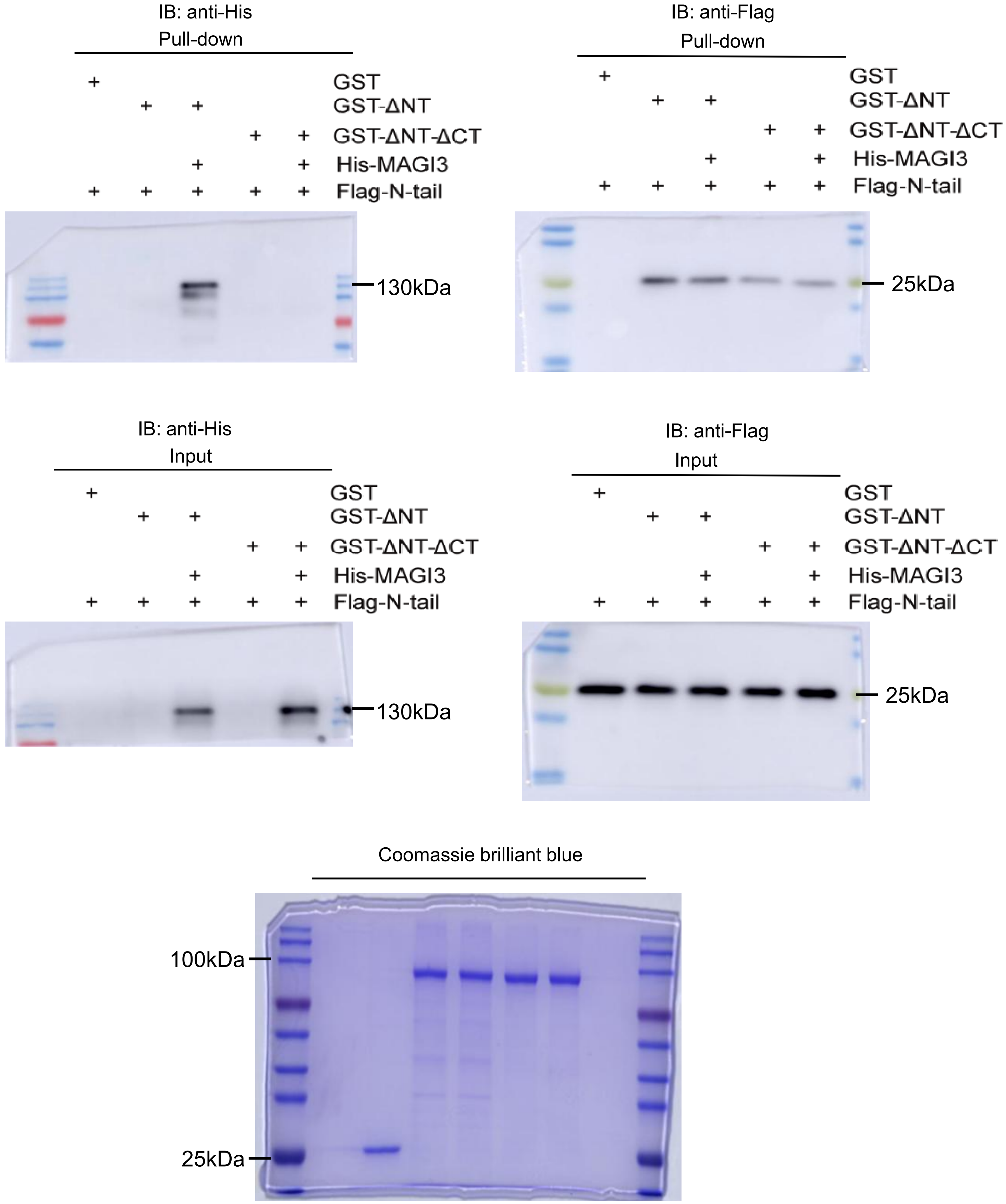

IB: anti-His
Pull-down
GST
GST-ΔNT
GST-ΔNT-ΔCT
His-MAGI3
Flag-N-tail
130kDa
IB: anti-Flag
Pull-down
GST
GST-ΔNT
GST-ΔNT-ΔCT
His-MAGI3
Flag-N-tail
25kDa
IB: anti-His
Input
GST
GST-ΔNT
GST-ΔNT-ΔCT
His-MAGI3
Flag-N-tail
130kDa
IB: anti-Flag
Input
GST
GST-ΔNT
GST-ΔNT-ΔCT
His-MAGI3
Flag-N-tail
25kDa
Coomassie brilliant blue
100kDa
25kDa

Figure S4

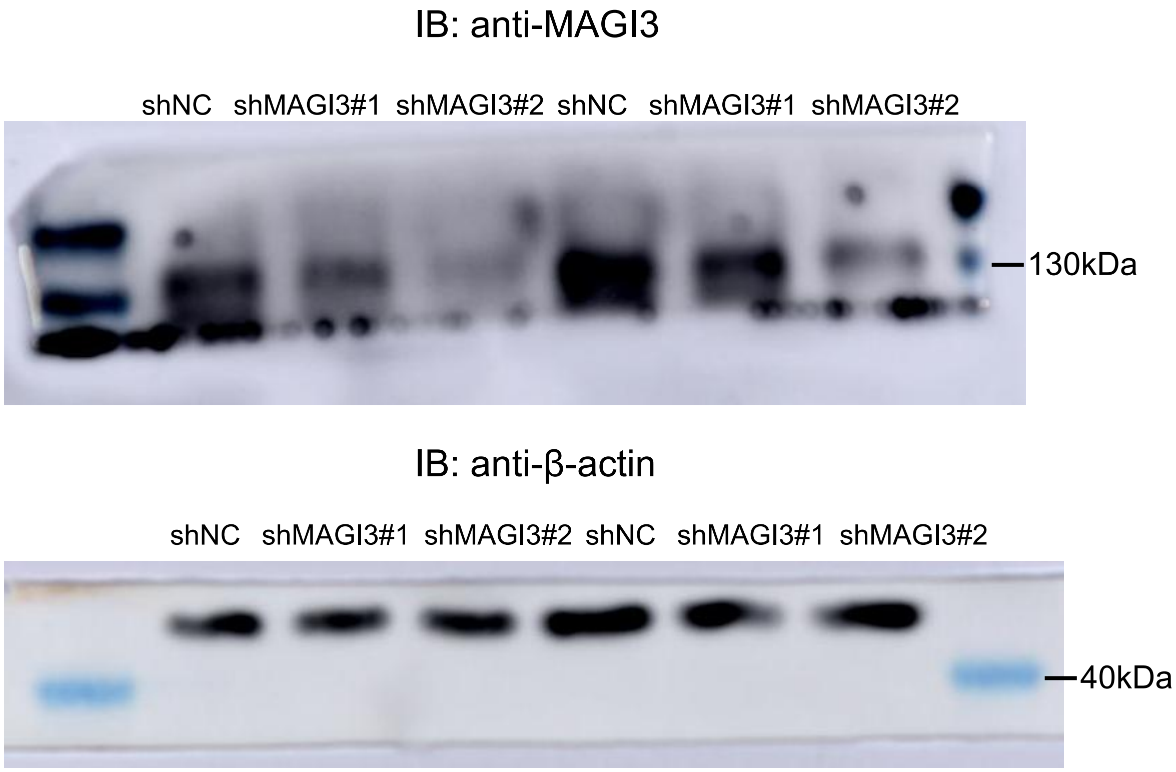

IB: anti-MAGI3
shNC shMAGI3#1 shMAGI3#2 shNC shMAGI3#1 shMAGI3#2
130kDa
IB: anti-β-actin
shNC shMAGI3#1 shMAGI3#2 shNC shMAGI3#1 shMAGI3#2
40kDa

Figure S7D

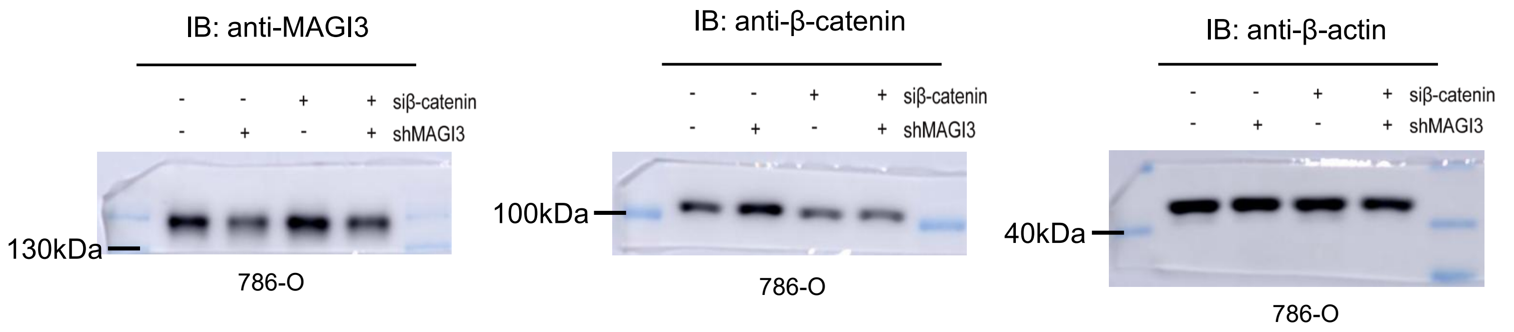

Figure S7E

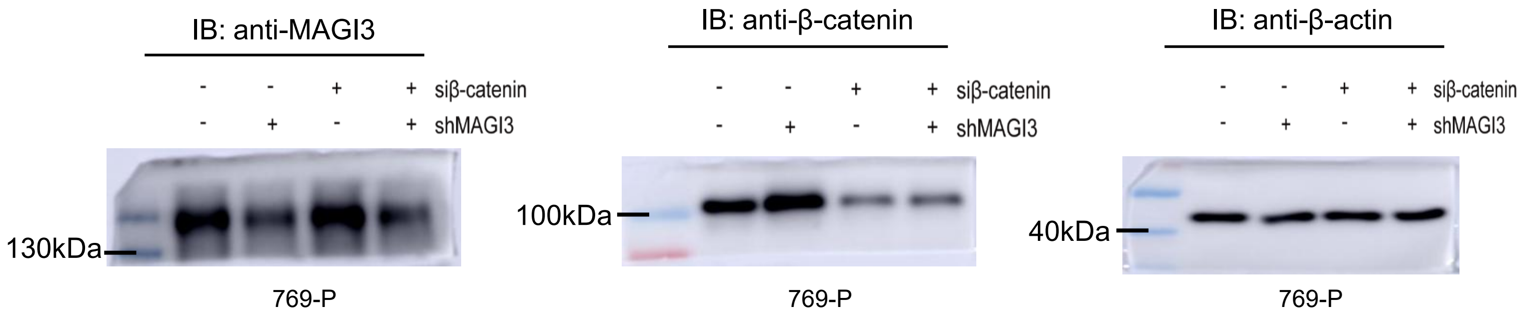

Supplement: Supplementary file 4 — uncropped western blots [file 41419_2026_8563_MOESM4_ESM.pdf]
